# Investigation of the methylerythritol 4-phosphate pathway for microbial terpenoid production through metabolic control analysis

**DOI:** 10.1186/s12934-019-1235-5

**Published:** 2019-11-05

**Authors:** Daniel Christoph Volke, Johann Rohwer, Rainer Fischer, Stefan Jennewein

**Affiliations:** 1 Fraunhofer Institute for Molecular Biology and Applied Ecology IME, Aachen, Germany; 2 Present Address: The Novo Nordisk Foundation Center for Biosustainability, Technical University of Denmark, Kongens Lyngby, Denmark; 3 Laboratory for Molecular Systems Biology, Department of Biochemistry, Stellenbosch University, Stellenbosch, South Africa; 4 Present Address: Purdue Institute of Inflammation, Immunology and Infectious Disease, Purdue University, West Lafayette, USA

**Keywords:** MEP pathway, Metabolic control analysis, Recombineering, Isoprene, *E. coli*

## Abstract

**Background:**

Terpenoids are of high interest as chemical building blocks and pharmaceuticals. In microbes, terpenoids can be synthesized via the methylerythritol phosphate (MEP) or mevalonate (MVA) pathways. Although the MEP pathway has a higher theoretical yield, metabolic engineering has met with little success because the regulation of the pathway is poorly understood.

**Results:**

We applied metabolic control analysis to the MEP pathway in *Escherichia coli* expressing a heterologous isoprene synthase gene (*isp*S). The expression of *isp*S led to the accumulation of isopentenyl pyrophosphate (IPP)/dimethylallyl pyrophosphate (DMAPP) and severely impaired bacterial growth, but the coexpression of *isp*S and isopentenyl diphosphate isomerase (*idi*) restored normal growth and wild-type IPP/DMAPP levels. Targeted proteomics and metabolomics analysis provided a quantitative description of the pathway, which was perturbed by randomizing the ribosome binding site in the gene encoding 1-deoxyxylulose 5-phosphate synthase (Dxs). Dxs has a flux control coefficient of 0.35 (i.e., a 1% increase in Dxs activity resulted in a 0.35% increase in pathway flux) in the isoprene-producing strain and therefore exerted significant control over the flux though the MEP pathway. At higher *dxs* expression levels, the intracellular concentration of 2-*C*-methyl-d-erythritol-2,4-cyclopyrophosphate (MEcPP) increased substantially in contrast to the other MEP pathway intermediates, which were linearly dependent on the abundance of Dxs. This indicates that 4-hydroxy-3-methylbut-2-en-1-yl diphosphate synthase (IspG), which consumes MEcPP, became saturated and therefore limited the flux towards isoprene. The higher intracellular concentrations of MEcPP led to the efflux of this intermediate into the growth medium.

**Discussion:**

These findings show the importance of Dxs, Idi and IspG and metabolite export for metabolic engineering of the MEP pathway and will facilitate further approaches for the microbial production of valuable isoprenoids.

## Highlights

- Metabolic control analysis of the MEP pathway in *E. coli.*
- Dxs has high flux and concentration control over MEP pathway intermediates.
- Metabolomic and proteomic analysis of the MEP pathway.
- Reduction of MEcPP to HMBPP is a limiting step for flux through the MEP pathway and promotes the efflux of MEcPP at higher flux rates.

## Background

Microbes can synthesize a broad spectrum of valuable compounds and precursors, providing an inexpensive and sustainable source of industrially-relevant chemicals including terpenoids. More than 60,000 terpenoids have been described [1], ranging from pharmacologically active molecules such as paclitaxel and artemisinin, which are used for the treatment of cancer and malaria, respectively [2, 3], to promising biofuels such as farnesene [4]. All terpenoids originate from the isomeric precursors dimethylallyl pyrophosphate (DMAPP) and isopentenyl pyrophosphate (IPP), both of which are produced via one of two natural metabolic routes: the 2-*C*-methyl-d-erythritol 4-phosphate (MEP) pathway and the mevalonate (MVA) pathway [5].

The MVA pathway has been engineered to produce large quantities of terpenoids [6, 7] but attempts to engineer the MEP pathway have been less successful [8, 9]. In part, this reflects our incomplete knowledge of the regulation and control of the MEP pathway, despite recent reports that have revealed a number of regulatory mechanisms [10–14]. Even so, the MEP pathway is a promising target for metabolic engineering because of its higher theoretical yield (~ 20% more than the MVA pathway under aerobic conditions in *Escherichia coli*) and its balanced use of reducing equivalents [15, 16]. Attempts to engineer the MEP pathway have thus far included randomization approaches, the combinatorial expression of a subset of MEP pathway genes [17–20] and the optimization of precursor supply [21]. Efforts to circumvent regulation by expressing the complete MEP pathway in heterologous hosts such as the yeast *Saccharomyces cerevisiae* have not been successful [22]. Several studies have investigated the rate-limiting steps in the MEP pathway, but have failed to deliver a consistent picture [17, 18, 20]. Others have found indications for the intrinsic regulation of the MEP pathway at the steps catalyzed by 1-deoxy-d-xylulose-5-phosphate synthase (Dxs) and 1-deoxy-d-xylulose-5-phosphate reductoisomerase (Dxr) [10], 2-C-methyl-d-erythritol-4-phosphate cytidyltransferase (IspD) [11] or 2-*C*-methyl-d-erythritol-2,4-cyclopyrophosphate synthase (IspF) [12]. The MEP pathway intermediate 2-*C*-methyl-d-erythritol-2,4-cyclopyrophosphate (MEcPP) is found at higher concentrations in the extracellular milieu in *E. coli* overexpressing MEP pathway genes [14]. However, the behavior of the MEP pathway has not been described in a precise and quantitative manner either in wild-type or engineered microbes.

We therefore set out to establish a quantitative description of the MEP pathway for terpenoid production using metabolic control analysis (MCA), a form of sensitivity analysis that quantifies the effect of small changes in a given parameter (such as enzyme activity) on overall system characteristics such as metabolite concentration and flux [23]. Here we used the MEP pathway for the production of isoprene. Besides having broad industrial applications, its low boiling point, and therefore its ease of purification, makes isoprene a promising product for fermentation process [9]. The MCA framework was applied to the concentrations of MEP pathway metabolites and the flux towards isoprene according to different expression levels of the *dxs* gene. To our knowledge, this is the first time that this approach was combined with recombineering, quantitative proteomics and metabolomics. This combination yielded a quantitative description of the control of Dxs on the flux through and metabolite concentrations in the MEP pathway, which will guide further engineering efforts.

## Materials and methods

### Bacterial strains and culture conditions

Standard cloning and metabolic engineering were carried out using *E. coli* strains 10β and BL21 (DE3), respectively (both supplied by New England Biolabs, Ipswich, MA, USA). For general cloning, the bacteria were cultivated in lysogenic broth (LB) medium, whereas for all other experiments the bacteria were cultivated in M9 medium supplemented with 0.5% (w/v) glucose and appropriate antibiotics (50 μg mL^−1^ kanamycin, 100 μg mL^−1^ ampicillin and/or 25 μg mL^−1^ chloramphenicol) at 37 °C [24]. Strains carrying the plasmids pSIM5 or pSIM6 [25] were grown at 30 °C and cured of the plasmids at 37 °C. Cultures were grown in baffled Erlenmeyer flasks filled to one-fifth of their nominal volume and agitated at 180 rpm. Cell growth in liquid medium was monitored by spectrophotometry to determine the optical density at 600 nm (OD_600_).

### General cloning and amplification

General cloning procedures and plasmid purification were carried out according to standard laboratory practice [24]. Herculase II Fusion DNA Polymerase (Agilent Technologies, Santa Clara, CA, USA) was used for the amplification of DNA fragments by the polymerase chain reaction (PCR) according to the manufacturer’s instructions. All plasmids and altered genomic regions were verified by Sanger sequencing carried out by Eurofins GmbH (Ebersberg, Germany).

### Construction of plasmids for the production of isoprene

The *isp*S gene from *Populus alba* was codon optimized for *E. coli* (Additional file 1: Table S1) (Thermo Fisher Scientific, Waltham, MA, USA) and the gene was amplified using forward primer 5′-AAT AAT TTT GTT TAA CTT TAA TAA GGA GAT ATA CC**A TG**G AAG CTC GTC GTT CTG C-3′ and reverse primer 5′-TTA GCG TTC AAA TGG CAG TAG CAA GCT TGT CGA CCA CGT TCG AAC GGC AGG ATC-3′ (start codon in bold). Vector pCOLA was amplified using forward primer 5′-GGT ATA TCT CCT TAT TAA AGT TAA ACA-3′ and reverse primer 5′-GGT CGA CAA GCT TGC GGC CG-3′. The products were joined by Gibson assembly yielding pCOLA::IspS, which was then amplified using forward primer 5′-GGC CGC ATA ATG CTT AAG TCG-3′ and reverse primer 5′-GCA AGC TTG TC GAC CTT AGC-3′. The isopentenyl diphosphate isomerase (*idi*) gene was amplified from the genome of *E. coli* strain BL21 using forward primer 5′-TAC TGC CAT TTG AAC GCT AAG GTC GAC AAG CTT GCA AGG AGA TAT ACC **ATG** CAA ACG GAA CAC GTC AT-3′ and reverse primer 5′-GAT TAC TTT CTG TTC GAC TTA AGC ATT ATG CGG CCT TAA TTG TGC TGC GCG AAA G-3′. The resulting fragments were again joined by Gibson assembly to yield pCOLA::IspS-idi.

#### Construction of dxs and dxr expression libraries

*Escherichia coli* was transformed with pSIM5 and grown at 30 °C to maintain the plasmid. Accordingly, we inoculated 20 mL LB medium with 200 μL of an overnight grown culture of *E. coli* pSIM5 and incubated the culture until the OD_600_ reached 0.5. The culture was then transferred to a water bath shaker of 42 °C and incubated for 10 min to induce expression of *gam*, *bet* and *exo*. The culture was then placed in ice slurry for 10 min. After centrifugation at 4000×*g* for 15 min at 4 °C, the supernatant was discarded and the pellet was resuspended in 20 mL double-distilled water at 0 °C and centrifuged again. This step was repeated twice and the pellet was then resuspended in 0.2 mL double-distilled water. We then added 100 pmol of the appropriate oligonucleotide: for the *dxs* expression library the sequence was 5′-GAC TAC ATC ATC CAG CGT AAT AAA TAA ACA ATA ACT DDD RRR RRD DDD CTG **ATG** AGT TTT GAT ATT GCC AAA TAC CCG ACC CTG GCA-3′, and for the *dxr* expression library the sequence was 5′-TCG AGC CGG TCG AGC CCA GAA TGG TGA GTT GCT T**CA T**GA AHH HHY YYY YHH TGA GAC AGA ATA AAA AGC AAA ACG CCG CCA GCC GAT CCG-3′ (changes from the genomic sequence are underlined). The oligomers were designed as described by Wang et al. [8] and contained four phosphorothioated bases at the 5′ terminus. A 50-µL aliquot of cells was used for electroporation and the cells were subsequently regenerated at 30 °C.

This procedure was carried out seven times with alternating use of pSIM5 and pSIM6, and the appropriate antibiotics. After the sixth round of recombineering, the cells were regenerated without antibiotics at 37 °C for 2 h and dilutions were plated. Single colonies were used for colony PCR. The genomic region containing the targeted mutation was amplified with forward primer 5′-ACC AGC AAC TTG GTA AAA GTA CC-3′ and reverse primer 5′-CGA TTT TGT CGC GGC G-3′ for the *dxs* expression library, and with forward primer 5′-ACA GCC AAC GCG CTT CAA TG-3′ and reverse primer 5′-TCG TGG TGA AGC AGA ACA AG-3′ for the *dxr* expression library. The amplicons were sequenced using the same primers.

### Metabolite quantification

A 10-mL volume of M9 medium supplemented with 0.5% (w/v) glucose was inoculated with 100 µL of overnight culture in 200-mL Erlenmeyer flasks. A 1-mL aliquot was withdrawn from the culture at OD_600_ ≈ 0.5 and centrifuged at 13,000×*g* for 1 min at 4 °C. The supernatant was discarded and the pellet was resuspended in 90 µL quenching solution at − 20 °C, before adding 10 µL 360 µM azidothymidine [26]. The quenching solution contained 40% methanol, 40% acetonitrile and 20% double-distilled water acidified with 0.5% formic acid [27]. The sample was incubated at − 20 °C for 1 h for quantitative extraction of MEP pathway intermediates. The solution was centrifuged at 17,000×*g* for 1 min at 4 °C and transferred to a measuring vial.

For the analysis of extracellular metabolites, 1 mL of culture was withdrawn and immediately centrifuged at 13,000×*g* for 1 min at 4 °C. A 20-µL aliquot of the supernatant was mixed with 70 µL of modified quenching solution (50% methanol, 50% acetonitrile acidified with 0.25% formic acid) and 10 µL 360 µM azidothymidine [26].

Calibration curves for absolute quantification were generated using analytical standards for all target metabolites. The standards were stored lyophilized at − 20 °C before preparing dilution series of mixed analytical standards in quenching solution. Calibration curves of intracellular metabolites were prepared by mixing 90 µL of each set of diluted standards with a lyophilized extract of 1 mL *E.* *coli* culture grown in minimal medium with U-^13^C glucose to OD_600_ = 0.5, to account for the matrix effects of other metabolites in *E. coli*. This step was skipped for the calibration curve of extracellular metabolites. We added 10 µL of the internal standard 360 µM azidothymidine. The solution was centrifuged at 17,000×*g* for 1 min at 4 °C and transferred to a measuring vial. To calculate the intracellular concentration from the concentration in the sample, an intracellular volume factor of 3.6 µL mL^−1^ OD_600_^−1^ was assumed [28].

The metabolites were analyzed using a Shimadzu (Tokyo, Japan) HPLC system coupled to a 6500 QTRAP mass spectrometer (Sciex, Darmstadt, Germany). The autosampler was cooled to 15 °C. The flow was held constant at 0.25 mL min^−1^. The oven was heated to 40 °C. The metabolites were separated according to the reversed phase ion pairing principle on a Nucleoshell RP18 column (2.7 µm, 90 Å, 100 mm) (Macherey–Nagel, Düren, Germany). Two buffers were used: Buffer A comprised 15 mM tributylamine and 20 mM formic acid, whereas buffer B was 100% methanol. The elution started with 0% buffer B for 2 min, followed by an increase to 40% buffer B over 1 min, a hold at 40% buffer B for 3 min, an increase to 100% buffer B over 6 min, then a decrease to 0% buffer B over 1 min, and a final hold for 4 min. Baseline separation of all intermediates other than IPP and DMAPP was achieved (Additional file 1: Fig. S1). The mass spectrometer was operated in negative mode with unit resolution for the mass filter Q1 and Q3 with optimized parameters for the HPLC method (Additional file 1: Table S2). The optimized parameters for each metabolite are listed in Additional file 1: Table S3.

### Protein quantification

We inoculated 50-mL of medium with 0.5 mL overnight culture and induced gene expression with 1 mM isopropyl β-d-1-thiogalactopyranoside (IPTG) at OD_600_ = 0.1. The culture was incubated until the OD_600_ reached 0.5 and then prepared as described by Gaida et al. [29].

#### In silico prediction of peptide mass and fragment size

In silico predictions and single reaction monitoring (SRM) screens were carried out using Skyline software [30]. After in silico digestion with trypsin, we excluded peptides outside the size range 8–20 amino acids, those containing cysteine residues or those with potential ragged ends due to tandem arginine and/or lysine residues. The remaining peptides were screened for double-charged species with single-charged y-series fragments after collision in collision cell Q2. Peptides and fragments with *m/z* values outside the range 50–1000 Da were excluded. The declustering potential and collision energy for all fragments were optimized in Skyline. Predicted transitions were sought in lysates of the *E. coli* BL21 strains overexpressing the corresponding gene. Proteotypic peptides were selected for each protein according to several criteria: (i) at least two transitions with high signal-to-noise ratios; (ii) retention times for all transitions equal and close to the predicted value [30]; (iii) transitions unique in the *E. coli* proteome, ensured by searching the NCBI database using BLAST [31] and Mascot [32]; and (iv) transition signal strength is orders of magnitude lower in the negative control not overexpressing the corresponding gene. If all criteria matched, proteotypic peptides were selected with the highest signal-to-noise ratio to achieve maximum sensitivity.

#### Synthetic internal standard and calibration curve

After selecting one proteotypic peptide for each protein of interest, precise quantities of each peptide were synthesized (JPT, Berlin, Germany) in normal and heavy forms, the latter containing ^13^C and ^15^N labeled lysine and arginine (SpikeTides L, JPT). The *m/z* values for detection of the labeled peptides were modified accordingly. All synthetic peptides included a C-terminal Qtag modification (JPT) that can be cleaved with trypsin. We used 1 nmol of heavy labeled peptides as an internal standard and this was introduced to the sample before reduction with dithiothreitol. The early introduction of the internal standard as well as the tag, which has to be cleaved off, ensures quantitative quality control throughout sample preparation and sample analysis. Known quantities of the synthetic peptides were used to establish a calibration curve based on the same preparation protocol used for all the other samples, including the addition of an internal standard. Again, an intracellular volume of 3.6 µL mL^−1^ OD_600_^−1^ was assumed [28].

#### Separation and detection of peptides

The peptides were separated by LC–MS/MS [33]. A 5-µL sample was injected into the Ascentis Express Peptide ES-C18 column (5 cm × 2.1 mm, 2.7 µm) (Sigma-Aldrich, St Louis, MO, USA) equipped with a compatible guard column. The peptides were eluted at a flow rate of 400 µL min^−1^ in 2% acetonitrile plus 98% double-distilled water containing 0.1% formic acid (buffer A) and 98% acetonitrile plus 2% double-distilled water containing 5% formic acid (buffer B). Elution began at 5% buffer B increasing to 40% buffer B in 17 min and then to 95% buffer B in 0.5 min, followed by a hold for 1 min before decreasing to 5% buffer B in 0.5 min and a hold for 3 min for re-equilibration. The peptides were quantified using Multiquant (Sciex, Darmstadt, Germany) according to the details provided in Additional file 1: Table S4. Baseline separation was achieved for all measured peptides (Additional file 1: Fig. S2).

### Isoprene quantification

*Escherichia coli* cultures were grown in M9 medium containing 0.1% (w/v) glucose in baffled Erlenmeyer flasks. At OD_600_ = 0.1, the cultures were induced with 1 mM IPTG. At OD_600_ = 0.5, several 1-mL aliquots of the culture were transferred to 10-mL vials and crimp-sealed. The sealed aliquots remained growing at 37 °C while shaking. At specific time points, vials were moved to boiling water and incubated for 5 min before cooling to 4 °C.

External calibration curves were used for quantification with a dilution series of commercial isoprene. All samples were incubated at 37 °C for at least 10 min before quantification. A heated syringe was used to withdraw 200 µL from the gas phase and to inject the sample into the heated (100 °C) injection port of the TQ8030 GC–MS/MS (Shimadzu). The samples were separated on a ZB-XLB-HT-Inferno capillary column (30 m × 0.25 mm, 0.25 µm) (Phenomenex, Aschaffenburg, Germany) with helium as the carrier gas. The temperature program began at 40 °C for 1 min, increasing linearly to 80 °C in 1 min followed by a hold for 1 min. Isoprene was detected in multiple reaction monitoring (MRM) mode with the transitions from 68.1 to 67 *m/z* and from 67.1 to 41 *m/z* with collision energies of 13 and 10 kV, respectively. The ion source was kept at 200 °C and the interface at 250 °C.

### Chemicals and reagents

Unless stated otherwise, all chemicals and inducers were purchased from Sigma-Aldrich. The following standards for the quantification of MEP pathway intermediates were supplied by Echelon Biosciences (Salt Lake City, UT, USA): 2-*C*-methyl-d-erythritol 4-phosphate (MEP), 1-deoxy-d-xylulose 5-phosphate (DXP), isopentenyl pyrophosphate (IPP) and dimethylallyl pyrophosphate (DMAPP). HPLC–MS-grade solvents were supplied by Carl-Roth (Karlsruhe, Germany) and U-^13^C-glucose was purchased from Cambridge Isotope Laboratories (Tewksbury, MA, USA). Gases for GC were purchased from Linde AG (Munich, Germany).

### Determination of flux by label incorporation

Fluxes in the MEP pathway in wild-type and mutants were calculated from the absolute ^13^C incorporation into DXP in time course labeling assays ranging from 10 s to 30 min. Overnight cultures of *E.* *coli* were used for inoculating of 40 mL of M9 minimal medium with appropriate antibiotics to an OD_600_ of ~ 0.02 AU. A temperature controlled vessel (custom-built, Ochs, Jena) was used for cultivation. The glucose concentration was adjusted to 0.05% with a 20% (w/v) glucose solution. This glucose concentration was calculated to be sufficient to allow the culture to grow to OD_600_ of 0.5 with residual glucose concentration high enough to prevent carbon starvation. The cultures were grown at 37 °C while stirred with a magnet to an OD_600_ of ~ 0.1 AU and induced with 1 mM IPTG. U-^13^C-glucose was added to a final concentration of 0.05% at an OD_600_ of ~ 0.5 AU. At chosen time points, 1 mL of culture was withdrawn and injected into a 10 mL 2% NaCl solution, kept at 0 °C to slow down further metabolism. The quenched culture was then immediately filtered through a 0.45-µm-filter (0.45 µm, 22 mm, PVDF, Merck Milipore) in a Swinnex filter holder (Merck Milipore). The filter was then transferred for extraction to a vessel with 1 mL 80% methanol [26], precooled to 0 °C. After an incubation time of 10 min, the extraction solution was transferred to a 2-mL-tube and the filter was extracted again and washed with 1 mL methanol. The extracts were merged and centrifuged at 15,000*g* for 1 min at 4 °C. The supernatant was transferred to a new tube and evaporated at 60 °C to dryness. The pellet was dissolved in 50 µL _dd_H_2_O and the metabolites were measured via LC–MS/MS. The calculation for non-stationary 13C-flux analysis, was adapted from Wright et al. [34]. The total labeled fraction of DXP was calculated using the *m*_*0*_ through *m*_*5*_ molecular ion species obtained from LC–MS/MS analysis according to the equation $1/N \mathop \sum \nolimits_{i = 1}^{N} M_{i} \times i$ [35], were *N* is the number of carbon atoms in the molecule and *M*_*i*_ is the fractional abundance of the *i*th isotopologue. The isotopologues are represented by *m*_*n*_ with *n* being the number of ^13^C atoms incorporated. Natural abundances of ^13^C, ^17^O, and ^18^O were measured in unlabeled standards and subtracted from labeled sample mass spectra to determine exact ^13^C amounts introduced during labeling. After plotting total fraction labeled against corrected labeling time, data were fitted to curves as the exponential rise to maximum, according to the equation $A \times \left( {1 - e^{{\left[ { - k \times t} \right]}} } \right)$, where *A* is the labeling plateau, *t* is the labeling time, and *k* is the kinetic rate constant. Equations were fitted to the time course data for each plant line by iteratively adjusting *A* and *k* to minimize $\chi^{2}$ values using the Levenberg–Marquardt minimization algorithm as implemented in the SciPy library of scientific computational routines (http://www.scipy.org). Seed estimates were obtained by visual inspection of the labeling maxima of the curves (for *A*), and by using $1/t_{1/2}$ (for *k*). The flux was then calculated for each line by multiplying the DXP pool size by the fitted rate constant *k*.

## Results and discussion

### Introducing isoprene synthase into *E. coli*

**Fig. 1:**
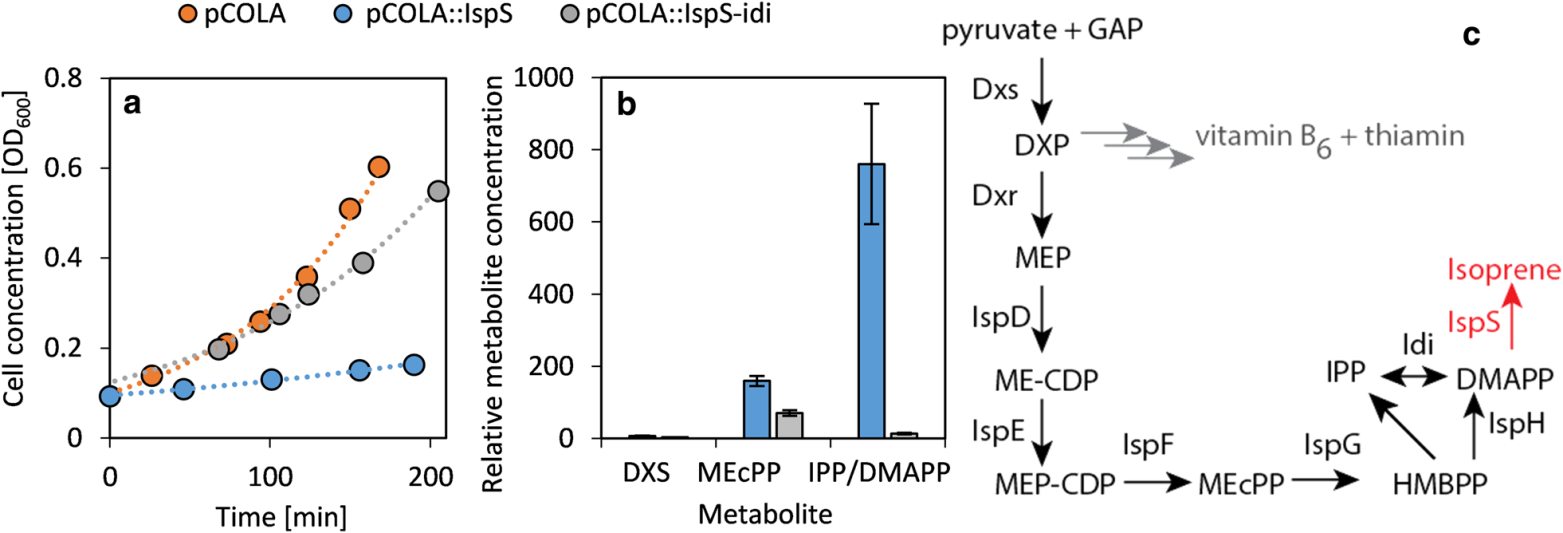
Expression of isoprene synthase (*isp*S) in *E. coli.* Strains carrying the empty plasmid pCOLA, the plasmid containing *isp*S (pCOLA::IspS) or the plasmid containing *isp*S and *idi* (pCOLA::IspS-idi) are compared. **a** Influence on *E.* *coli* growth. *E.* *coli* carrying the empty plasmid shows normal exponential growth kinetics. **b** Influence on MEP pathway intermediate concentrations. The concentration is shown relative to wild-type bacteria carrying the empty vector pCOLA. The data represent the mean of biological triplicates ($\overline{\varvec{x}}$± SE; n = 3). **c** The MEP pathway in *E. coli*. The metabolite DXP is also a precursor for the synthesis of vitamin B_6_ and thiamin. Ispoprene can be synthesized through the heterologous expression of *isp*S (shown in red). *DXP* 1-deoxy-d-xylulose 5-phosphate, *MEP* 2-*C*-methyl-d-erythritol 4-phosphate, *MEcPP* 2-*C*-methyl-d-erythritol 2,4-cyclopyrophosphate, *ME-CDP* 4-diphosphocytidyl-2-*C*-methylerythritol, *MEP-CDP* 4-diphosphocytidyl-2-*C*-methyl-d-erythritol 2-phosphate, *HMBPP* 4-hydroxy-3-methyl-but-2-enyl pyrophosphate, *IPP* isopentenyl pyrophosphate, *DMAPP* dimethylallyl pyrophosphate, *GAP* glyceraldehyde 3-phosphate, *Dxs* DXP synthase, *Dxr* DXP reductoisomerase, *IspD* MEP cytidylyltransferase, *IspE* ME-CDP kinase, *IspF* MEcPP synthase, *IspG* HMBPP synthase, *IspH* HMBPP reductase, *Idi* isopentenyl diphosphate isomerase, *IspS* isoprene synthase

**Fig. 2:**
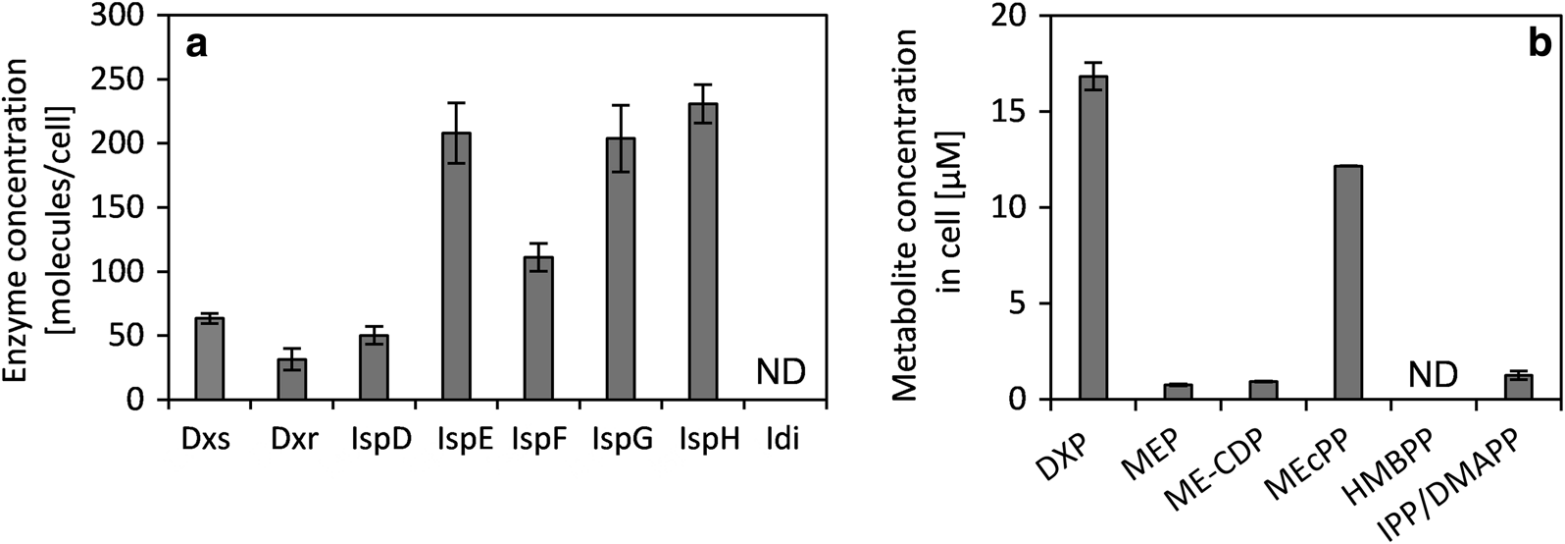
**a** Concentration of MEP pathway enzymes in *E.* *coli*. Idi was not detected. **b** Concentration of MEP pathway intermediates in *E.* *coli*. MEP-CDP was not quantified because it is highly unstable, and HMBPP was not detected. ($\overline{\varvec{x}}$± SD; n = 3)

We chose to synthesize isoprene, an important platform chemical, as a model terpenoid in *E. coli* as it has several advantages over other terpenoids. Its synthesis in microbes requires only the expression of one heterologous gene, *ispS*. Furthermore, its high volatility reduces toxicity, facilitates product recovery and reduces feedback inhibition by the product [9]. We employed the *ispS* gene from *Populus alba*, because it has been used in many microbial engineering processes and is known to express well [7, 9, 20]. We analyzed the *E. coli* strain overexpressing the *isp*S gene under the control of the T7 promoter through quantitative proteomics and metabolomics. The induction of *isp*S expression strongly inhibited bacterial growth (Fig. 1a). Metabolomic characterization revealed a strong increase in the concentration of IPP/DMAPP and also higher concentrations of MEcPP (Fig. 1b). Because IspS produces isoprene from DMAPP via the elimination of diphosphate (Fig. 1c), we speculated that the catalytic activity of Idi (converting IPP to DMAPP) was too low to keep up with the consumption of DMAPP by IspS, which would result in low concentrations of DMAPP. Most enzymes that consume IPP also need DMAPP as a substrate, so the production of native terpenoids would be inhibited and the excess IPP would remain unconsumed. A feedback inhibition of Dxs by IPP and DMAPP by DMAPP/IPP as in *Populus trichocarpa* [13] would enhance this effect. The proposed low specific activity of Idi was further supported by proteomic analysis of the MEP pathway in *E.* *coli*. Here all of the MEP pathway enzymes except of Idi were detected, meaning that less than 10 copies of Idi per cell are present, given the limit of detection (Fig. 2). This is consistent with the *idi* gene being non-essential as confirmed by the normal growth of the *idi* mutant, in contrast to the other MEP pathway genes [36]. Tests involving the randomization of the *idi* ribosome binding site (RBS) to change the expression level had no effect on the abundance of the enzyme or the amount of isoprene produced (data not shown). We hypothesise that it is likely that the ratio of IPP to DMAPP synthesized by (E)-4-hydroxy-3-methylbut-2-enyl diphosphate reductase (IspH), which is 5:1 in *E.* *coli* [37], evolved to match the ratio needed for terpenoid synthesis and therefore *idi* is not expressed or expressed at low levels under normal conditions. Consequently, modulation of the translation rate of *idi* through mutation of the RBS has little effect on the expression. Expression of *idi* might be induced under conditions, where there is an increased demand for specific terpenoid products.

The specific activity of Idi was increased by overexpression of a plasmid-borne *idi* gene under the control of the T7 promoter, provided by the vector pCOLA::IspS-idi. The coexpression of *idi* and *isp*S overcame the growth impairment observed with *isp*S alone, and restored the native levels of IPP/DMAPP (Fig. 1b). For all further experiments, *idi* was coexpressed with *isp*S in order to prevent the isomerase from limiting the flux or inhibiting bacterial growth.

### Proteomic and metabolomic analysis of the MEP pathway

The maximum reaction rate per cell (*V*_max_), the reaction rate under substrate saturation, of the enzymes in vivo was calculated by multiplying the protein abundance (Fig. 2) with its turn over number (k_cat_) from literature. The values lay between 4.8 × 10^3^ (IspG) and 1.8 × 10^7^ (Dxr) molecules cell^−1^ min^−1^ as shown in Table 1. The turnover numbers of IspG and IspH were measured using artificial reducing agents [49, 50] and it is unclear how these conditions relate to those in vivo.

**Table 1:** Catalytic power of the MEP pathway enzymes in *E. coli*

Enzyme	*k*_cat_ (10^3^ min^−1^)	References for *k*_cat_	Amount per cell	Maximum reaction rate per cell (*V*_max_) (10^3^ molecules min^−1^ cell^−1^)
Dxs	0.25	[41]	64 ± 4	16
Dxr	1.3; 6.7; 5.9 × 10^2^	[42–44]	32 ± 5	41; 2.1 × 10^2^; 1.8 × 10^4^
IspD	2.94	[47]	50 ± 8	1.48 × 10^2^
IspE	1.01^b^	[48]	208 ± 22	2.1 × 10^2^
IspF	0.06		111 ± 13	6.66
IspG	0.024^a^	[49]	204 ± 25	4.83
IspH	1.13^a^	[50]	218 ± 22	2.46 × 10^2^
Idi	0.0048–0.02	[36, 51]	< 10	< 0.2

^a^ Kinetics were partly determined using artificial reducing agents

^b^ Calculated from a *V*_max_ of 162.9 nmol min^−1^ for 5 µg pure IspE and using 31 kDa as the molecular weight of IspE

The enzymatic steps catalyzed by Dxs and Dxr lie behind the branching points of the MEP pathway: glyceraldehyde phosphate (GAP) and pyruvate are glycolytic intermediates, DXP is the substrate for the synthesis of pyridoxal [38] and thiamine phosphate [39]. Therefore, the concentrations of these intermediates depend not only on the MEP pathway, but also on enzymes outside of the MEP pathway and will therefore probably not result in substrate saturation of these enzymes. In contrast, the remaining MEP pathway intermediate pools (MEP, ME-CDP, MEP-CDP, MEcPP and HMBPP) only depend on the activities of the MEP pathway enzymes responsible for their synthesis and subsequent conversion. Therefore, these concentrations can be correlated to the flux through the MEP pathway, and substrate saturation of the consuming enzyme is then possible. Hence, these enzymes could in principle reach *V*_max_. The reaction rate (*v*) of Dxs and Dxr can be calculated based on the substrate concentrations. The concentration of any cofactors is ignored in this consideration. The intracellular concentration in *E. coli* of GAP is 10.3 µM [40], which is 4.6% of the Michaelis constant (*K*_m_) of Dxs (226 µM according to Brammer and Meyers [41]) resulting in a *v* of 3.7 × 10^2^ molecules cell^−1^ min^−1^. The reaction rate of Dxr lies between 4.9 × 10^2^ and 8.4 × 10^5^ molecules cell^−1^ min^−1^ depending on which literature values are used for the calculation (Additional file 1: Table S5) [42–44]. Therefore, the reaction rate of Dxs is predicted to be the lowest among the MEP pathway enzymes, apart from Idi (Table 1). Therefore, Dxs potentially exerts a high flux control on the MEP pathway [45]. Furthermore, it occupies a key position in the pathway [45]. Hence, Dxs was chosen for further analysis. The intracellular concentration of GAP is much lower than the *K*_m_ of Dxs, which explains why manipulating the GAP concentration strongly influences the flux through the MEP pathway [21, 46].

The major intermediate pools of the MEP pathway are DXP and MEcPP (Fig. 2b). For all enzymes, the measured substrate concentrations lay far below their K_m_ values (Table 2). As a consequence, the flux is highly adaptable to substrate availability, as the flux can be strongly increased when substrate accumulates or reduced when substrate concentration falls [52]. In contrast, the activity of enzymes, which are not on branching points, exerts little control on the flux, allowing the pathway to be regulated by its first enzyme, Dxs. Furthermore, this sub-maximal flux gives the pathway a reserve flux capacity, which is the difference between the flux with the given substrate concentration (K_m_ ≫ substrate concentration) and the maximum flux under substrate saturation (K_m_ ≪ substrate concentration). This reserve flux capacity allows for a quick increase in flux without the need of changes in gene expression [53].

**Table 2:** Concentration of MEP intermediates compared to the *K*_m_ values of the downstream enzymes

Enzyme	*K*_m_ (µM)	Concentration in *E.* *coli* (µM)
Dxs	226 GAP^a^	10.3^b^
Dxr	99^c^; 115^d^; 720^e^ DXP	16.8 ± 0.7
IspD	61^f^; 370^g^ MEP	0.75 ± 0.04
IspE	150^h^ ME-CDP	0.92 ± 0.02
IspG	311^i^; 560–700^j^ MEcPP	12.2 ± 0.01
Idi	~ 9.5/14.3^k^ IPP/DMAPP	1.3 ± 0.2

^a^ [41]; ^b^ [40]; ^c^ [42]; ^d^ [43]; ^e^ [44]; ^f^ [48]; ^g^ [47]; ^h^ [54]; ^i^ [49]; ^j^ [55]; ^k^ [36]

### Construction and evaluation of a *dxs* expression library

**Fig. 3:**
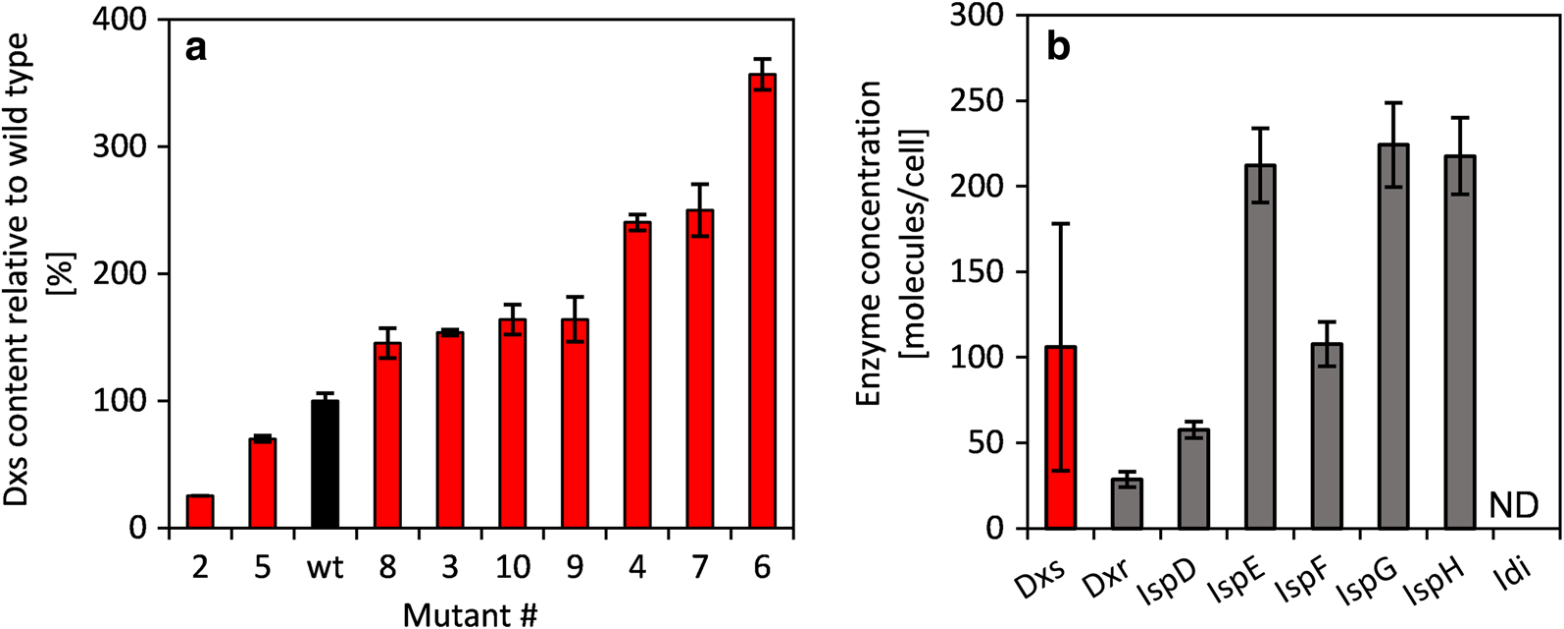
Expression differences in the *dxs* expression library. **a** Dxs content relative to wild-type. Data are means of biological triplicates ($\overline{\varvec{x}}$± SD, n = 3). **b** Concentration of MEP pathway enzymes. Clones 1–10 were analyzed ($\overline{\varvec{x}}$± SD, n = 30)

A *dxs* expression library was constructed to investigate the control exerted by Dxs on the flux through the MEP pathway. In order to minimize alterations to the genome and hence to the overall metabolism, recombineering was used to mutate the RBS of *dxs*. The benefit of this approach is that no polarity effects need to be considered and the plasmid carrying the genetic tools can be removed by curing before further experiments, leaving a near-wild-type genome containing only the desired mutation. After seven cycles of recombineering, the region containing the *dxs* RBS was sequenced, revealing that ~ 40% of the clones contained the desired randomization (Additional file 1: Table S6). Proteomic analysis revealed changes in the Dxs content of the mutant strains, ranging from 25 to 357% (Fig. 3a). Nine mutants and the wild-type were transformed with pCOLA::IspS-idi. Importantly, the concentration of all other MEP pathway enzymes showed no significant changes (Fig. 3b). This allowed the analysis of Dxs isolated from other components of the MEP pathway. Likewise, a *dxr* expression library was constructed (Additional file 1: Table S7), but despite the heterogeneous expression levels (Additional file 1: Fig. S3), the mutations did not lead to changes in isoprene production (data not shown) and no further evaluations were carried out.

### Isoprene emission in the *dxs* expression library

**Fig. 4:**
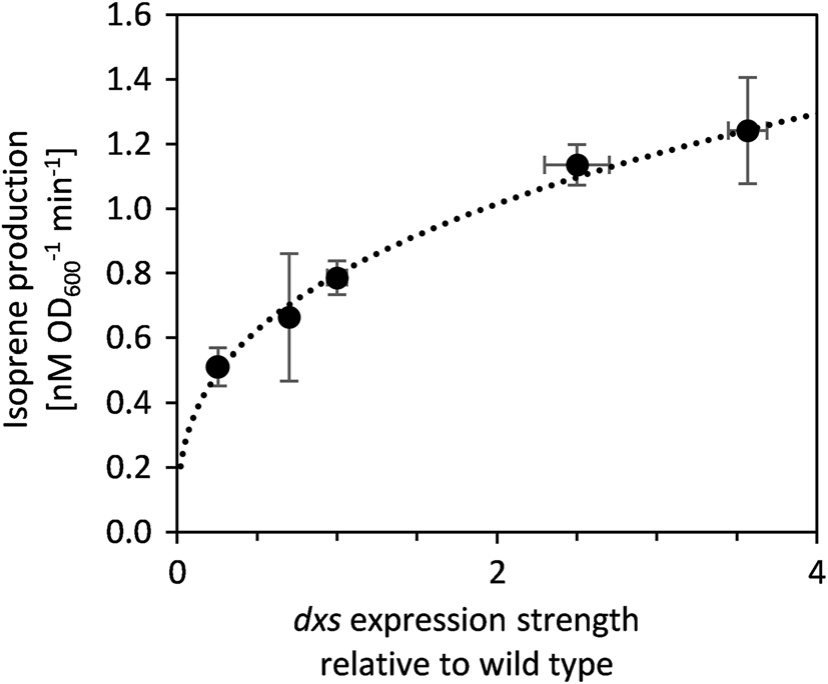
Isoprene production in engineered *E.* *coli* strains with altered *dxs* expression levels. The dotted line represents a fit of the data to the power function 0.8 × ^0.35^ While the fit was performed on all data points individually, the data are visualized as $\overline{\varvec{x}}$± SD (n = 3) for clarity

The impact of changes in Dxs concentration on isoprene production in the strain expressing the isoprene synthase was evaluated under aerobic growth conditions. Because isoprene is highly volatile (boiling point = 36 °C), assays were conducted in sealed flasks with sufficient oxygen to allow aerobic growth until measurement. In order to assess the productivity over time, the start and end production levels were measured precisely by sealing the culture at the specific time point and inactivating the culture by a sudden increase in temperature. Isoprene production per unit time and biomass was constant over the duration of the experiment (Additional file 1: Fig. S4), indicating that the in vivo conditions remained constant during incubation. In the strain expressing a plasmid encoded isoprene synthase and *idi* gene, isoprene production as a function of *dxs* expression was well fitted by the function 0.8 × ^0.35^ (Fig. 4). The control coefficient was 0.35 at wild-type *dxs* expression levels (Additional file 1: Fig. S5). At higher *dxs* expression levels, isoprene production saturated and Dxs therefore exerted less control over isoprene productivity. This is in accordance with the theory that flux control in the pathway is shared, and is shifted towards other enzymes if the activity of one enzyme increases [56]. The saturation effect was already pronounced when Dxs concentration had increased by less than twofold, suggesting that further increase in *dxs* expression levels are likely to have only a minor additional impact on isoprene production. This is in agreement with the observation that severe overexpression of *dxs* under the control of the T7 promoter increases isoprene production by about threefold in batch fermentation (Additional file 1: Fig. S6). The effect of *dxs* overexpression on terpenoid production was similar to the range reported in other studies [8, 17, 18].

### Metabolic characterization of the MEP pathway in the *dxs* expression library

The concentrations of the MEP pathway intermediates were determined in the *dxs* expression library expressing *isp*S and *idi* from pCOLA::IspS-idi. The intermediate MEP-CDP was not quantified due to its instability. The isomers IPP and DMAPP were quantified together. HMBPP was present at levels below the limit of detection in all samples (0.39 µM). Remarkably, the expression of *isp*S and *idi* led to a significant increase in the levels of all measured metabolites except MEcPP (Additional file 1: Table S8). We speculate that this effect might be due to a feedback inhibition by a metabolite further downstream in the terpenoid biosynthesis pathway, whose concentration is reduced through the diversion of the carbon flux into isoprene.

**Fig. 5:**
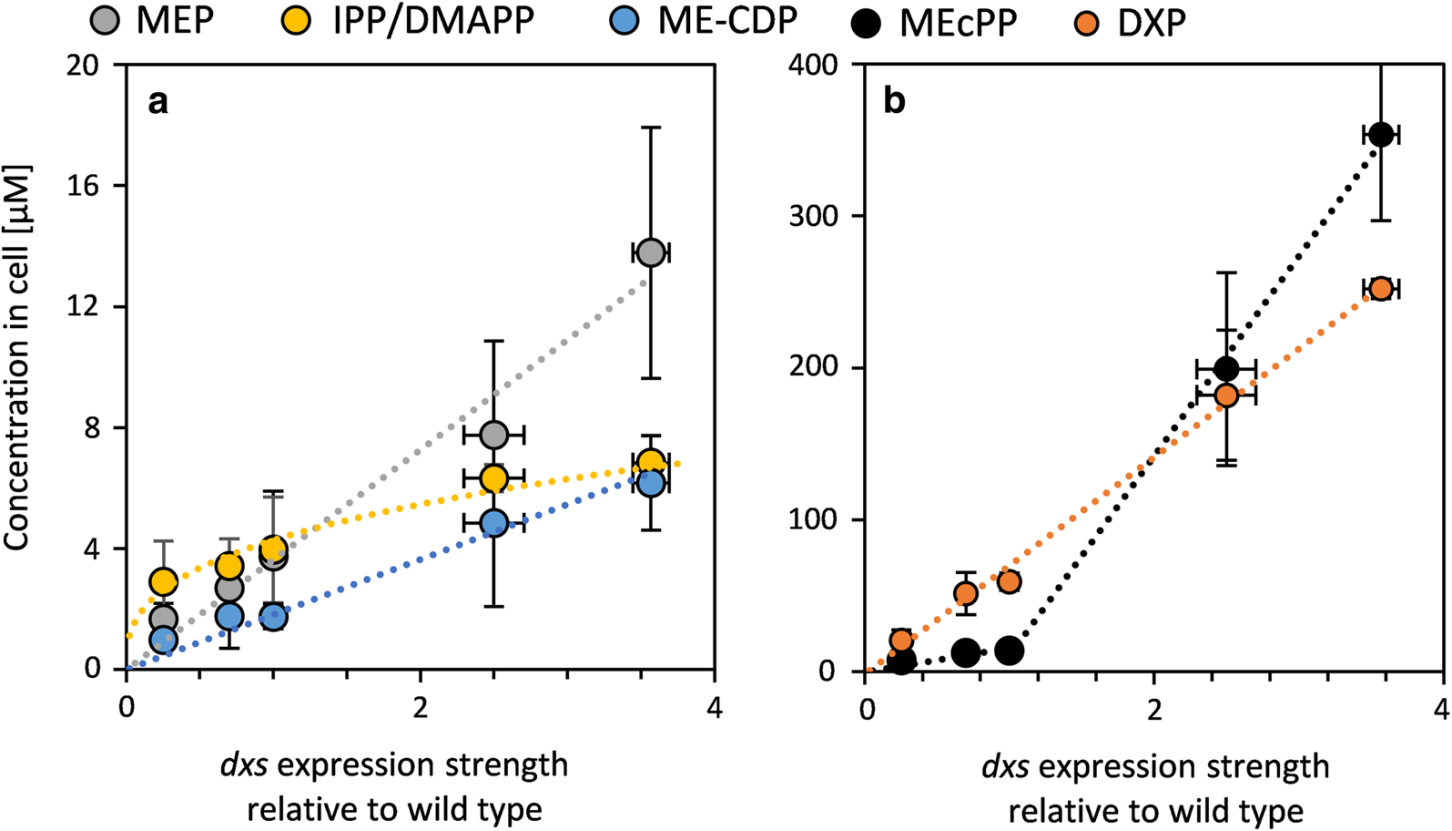
Concentration of MEP, IPP/DMAPP, and ME-CDP (**a**), and MEcPP and DXP (**b**) in response to changes in *dxs* expression ($\overline{\varvec{x}}$± SD, n = 3). The graphs for MEP, ME-CDP and DXP are fitted with a linear fit through zero, while IPP/DMAPP is fitted with a power law (R^2^ > 0.95). The intermediate concentrations were measured in the *dxs* RBS library expressing *isp*S and *idi* from pCOLA::IspS-idi. The cultures were induced at OD_600_ = 0.1 and the intermediates were quantified at OD_600_ ≈ 0.5. The concentration of MEcPP in response to *dxs* expression was fitted to two piecewise linear segments. The graph is divided at the wild-type expression level, with overexpression yielding a slope more than 14-fold steeper than under expression. The metabolite concentration control coefficient of Dxs over MEcPP for the overexpression of *dxs* is 2.6. Other concentration-control coefficients are given in Additional file 1: Table S9 and were calculated according to Additional file 1: Fig. S8, the double logarithmic plot of the data in this figure

The concentration of all intermediates was linearly dependent on the concentration of Dxs at wild-type expression levels and below (Fig. 5a, b), whereas higher concentrations of Dxs caused a massive increase in the levels of MEcPP (Fig. 5b), while the levels of IPP/DMAPP approached saturation (Fig. 5a). These characteristics indicate that as IspG reached substrate saturation and approached its V_max_, the effect of higher substrate concentrations on the reaction rate decreased. Furthermore, we suspected that not all the flux entering the MEP pathway was reaching IPP/DMAPP, which results in a less-than-expected increase in IPP/DMAPP concentrations. This diverted flux was later confirmed to be metabolite export. The levels of DXP, MEP and ME-CDP were linearly dependent on *dxs* expression, whereas levels of IPP/DMAPP were best fitted by the power law with the equation 4.3 × ^0.35^. Notably, the IPP/DMAPP concentration had the same exponent as isoprene production, which leads to a linear dependency of these values (Additional file 1: Fig. S7). The metabolite concentration control coefficient of Dxs decreased with increasing distance in the pathway to the metabolite pool (Additional file 1: Table S9).

### Flux through DXP

The direct quantification of all products of the MEP pathway is difficult, as a large variety of different terpenoids is produced, of which some are conjugated to other molecules, for example in the prenylation of proteins. Therefore, non-stationary ^13^C flux analysis was used to assess the flux through the MEP pathway. Here, the information about time-dependent label incorporation into the metabolite pools of DXP was used to quantify the carbon flux through the pathway (Additional file 1: Fig. S9).

**Fig. 6:**
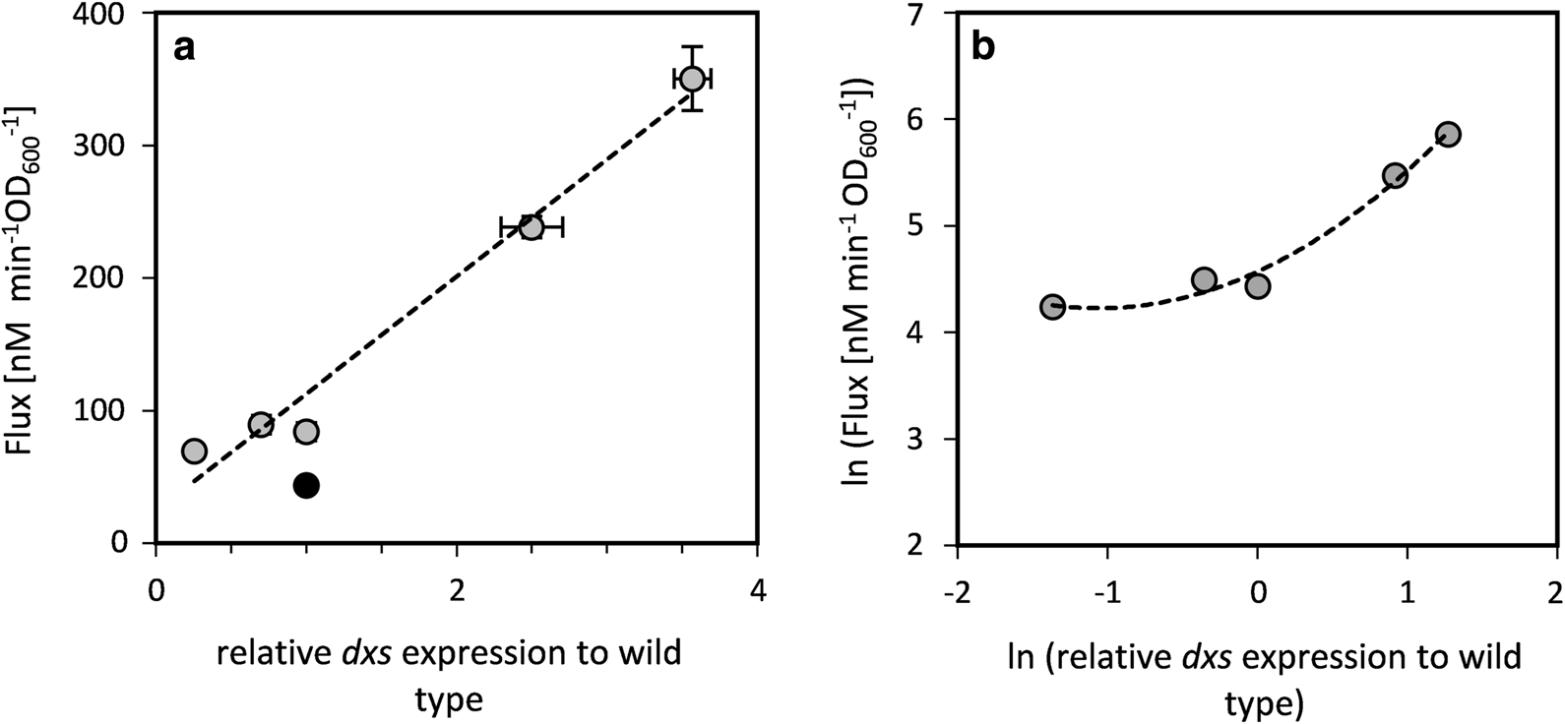
**a** Dependence of carbon flux through the DXP pathway on *dxs* expression in *E.* *coli* wild type carrying the empty plasmid pCOLA (•) and in the strain expressing *ispS* and *idi* from the plasmid pCOLA::IspS-idi (•). The data was fitted by linear regression. **b** Logarithmic plot of the flux through DXP as a function of *dxs* expression. The data was fitted with a second-degree polynomial function

The flux through the MEP pathway increased with increased expression levels of *dxs* in *E. coli* expressing *ispS and idi* from the plasmid pCOLA::IspS-idi (Fig. 6a). The flux control coefficient of Dxs on the flux through DXP (0.65) is very high (Fig. 6b), meaning that the flux changes 0.65% with every 1% change in Dxs activity. The summation theorem of MCA states that the flux control coefficients of all enzymes in a metabolic pathway sum to one [23]. This suggests that Dxs is the major flux-controlling enzyme of the MEP pathway. Furthermore, a higher flux through DXP was observed upon expression of *IspS* and *Idi* compared to the wild type carrying the empty plasmid (Fig. 6a). This increase could be either due to an activation of the pathway by IspS, or because Idi: IspS consumes DMAPP, which is known to inhibit Dxs, the first enzymatic reaction of the MEP pathway [13]. Idi could increase the flux through balancing the pool of DMAPP and IPP and thereby accelerate downstream reactions.

Even though the control of Dxs on the isoprene production saturated (Fig. 4), the flux did not. This indicates that the flux was directed into other sinks at higher *dxs* expression (e.g. terpenoids and exported metabolites). This also agrees with the observation that the flux control coefficient on the early pathway metabolite, DXP, is higher than on isoprene, as part of the flux does not reach isoprene. The observed flux through DXP was several times higher than the sum of DXP and MEcPP export and isoprene emission, which could be due to the production of endogenous terpenoids.

### Export of MEP pathway intermediates

**Fig. 7:**
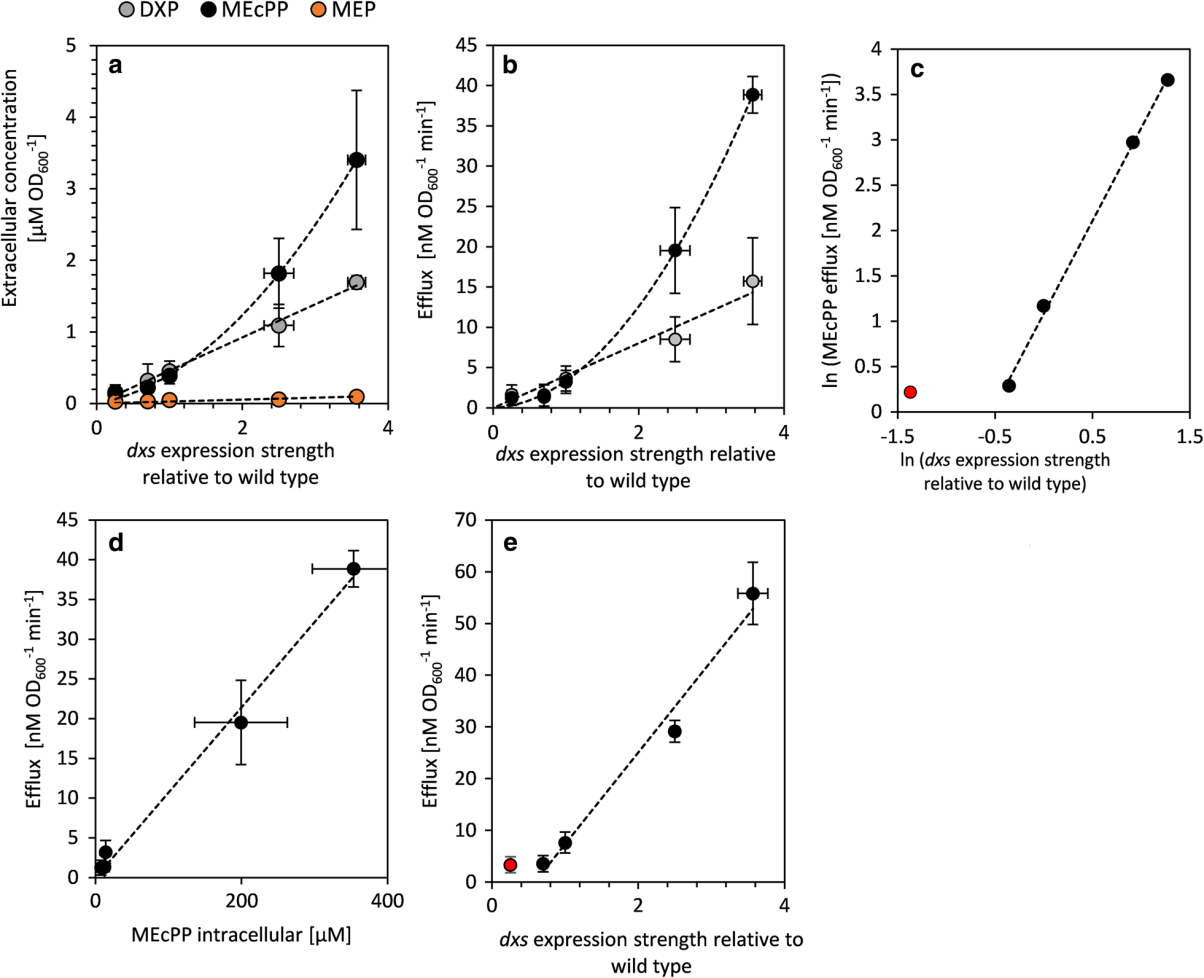
Specific efflux rates of MEP pathway intermediates calculated from their measured concentrations in the supernatant of cultures of the *E.* *coli* Bl21 *dxs* RBS library expressing *isp*S and *idi* from pCOLA::IspS-idi ($\overline{\varvec{x}}$ ± SD, n = 3). The cultures were induced at OD_600_ = 0.1 and the supernatant was sampled at OD_600_ ≈ 0.5. The export of MEP pathway intermediates occurred after the induction of *isp*S expression in the *dxs* expression library. **a** Metabolite concentration in the supernatant of the cultures. **b** DXP and MEcPP efflux rates as a function of *dxs* expression relative to wild-type levels. Linear fits through zero and second-degree polynomial fits through zero are shown for DXP and MEcPP, respectively in (**a**) and (**b**). **c** Logarithmic plot of MEcPP efflux rate as a function of *dxs* expression. The data were fitted to a linear function with a slope of 2.0. The red marked data point was excluded from the fit. **d** The correlation of MEcPP efflux rate with its intracellular concentration, linear fitted through zero. **e** Combined efflux of DXP, MEcPP and isoprene in dependents of *dxs* expression, linear fitted

To determine whether part of the flux leaves the MEP pathway before reaching IPP/DMAPP, we analyzed the culture supernatants. MEP, DXP and MEcPP were detected in the spent medium of cultures producing isoprene (Fig. 7a). The export rates of DXP and MEcPP into the supernatant increased as *dxs* expression levels increased (Fig. 7b). MEP was detected in the supernatant, but concentrations were too low for kinetic measurements. The export rate of DXP increased linearly with increasing *dxs* expression, while the increase in MEcPP efflux rate into the supernatant was more than linear (Fig. 7b, c). The MEcPP efflux rate was, however, proportional to the intracellular MEcPP concentration (Fig. 7d). This supports the hypothesis that MEcPP is exported rather than reduced to HMBPP as a consequence of the flux into the MEP pathway being higher than the *V*_max_ of IspG. The total flux towards extracellular DXP, MEcPP and isoprene increased drastically with increased expression of *dxs* (Fig. 7e).

## Conclusions

**Fig. 8:**
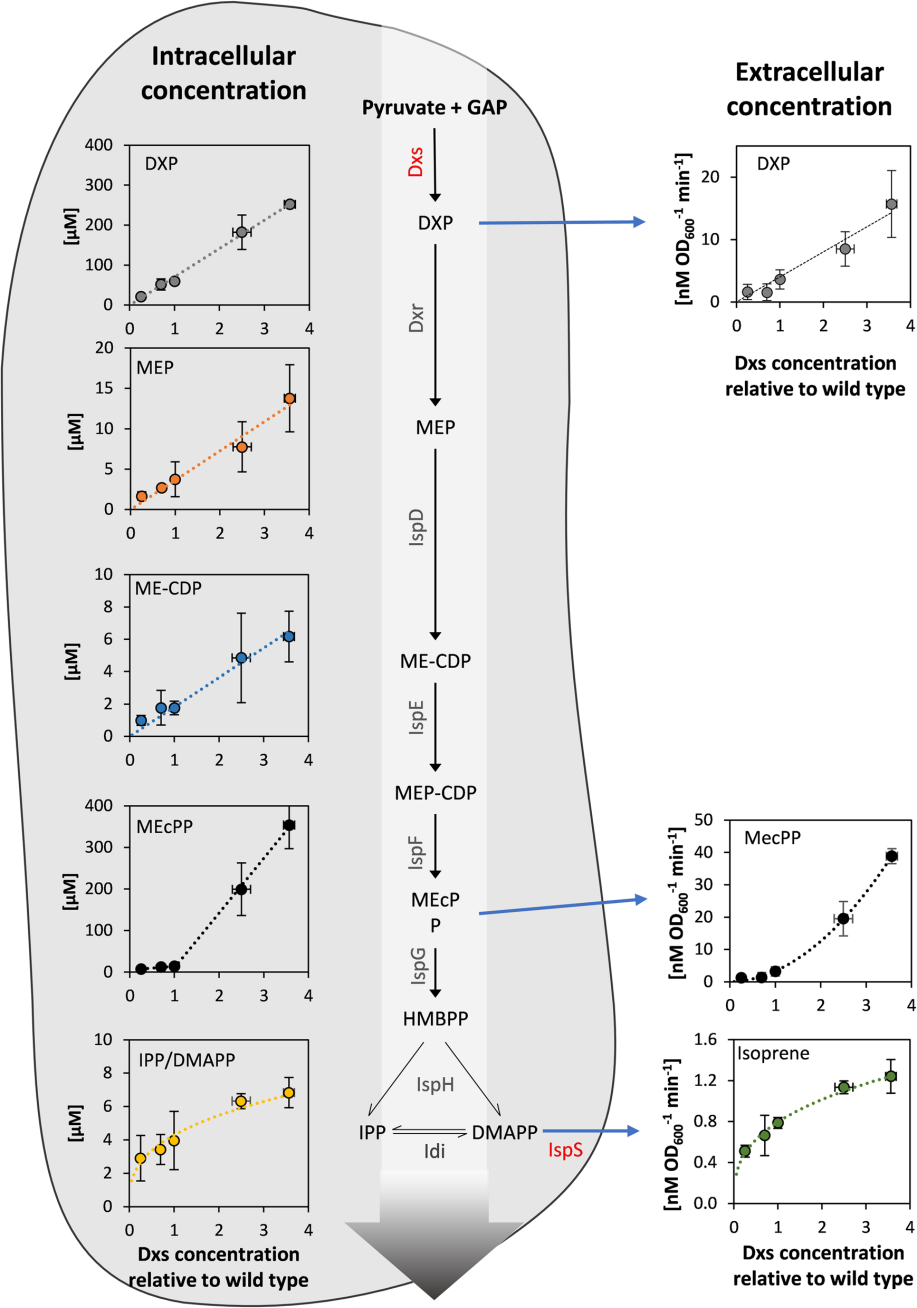
Metabolic characterization of the MEP pathway in *E. coli* expressing isoprene synthase and isopentenyl pyrophosphate isomerase from a plasmid. The intracellular metabolite concentrations, secretion rate of metabolites and isoprene production rate are plotted against *dxs* expression. The expression strength of *dxs* was modulated by randomization of its ribosome-binding site. *DXP* 1-deoxy-d-xylulose 5-phosphate, *MEP* 2-*C*-methyl-d-erythritol 4-phosphate, *MEcPP* 2-*C*-methyl-d-erythritol 2,4-cyclopyrophosphate, *ME-CDP* 4-diphosphocytidyl-2-*C*-methylerythritol, *MEP-CDP* 4-diphosphocytidyl-2-*C*-methyl-d-erythritol 2-phosphate, *HMBPP* 4-hydroxy-3-methyl-but-2-enyl pyrophosphate, *IPP* isopentenyl pyrophosphate, *DMAPP* dimethylallyl pyrophosphate, *GAP* glyceraldehyde 3-phosphate, *Dxs* DXP synthase, *Dxr* DXP reductoisomerase, *IspD* MEP cytidylyltransferase, *IspE* ME-CDP kinase, *IspF* MEcPP synthase, *IspG* HMBPP synthase, *IspH* HMBPP reductase, *Idi* isopentenyl diphosphate isomerase, *IspS* isoprene synthase

The metabolic engineering of the MEP pathway in microbes has significant potential for the industrial production of terpenoids, but this approach has not been as successful as anticipated mainly because the regulation of the MEP pathway is not fully understood. Here, we investigated the behavior of the MEP pathway in metabolically engineered *E. coli*, producing the model terpenoid isoprene. Firstly, we have confirmed that the overexpression of *idi* increases the growth rate of the isoprene production strains and increases the isoprene production rate, making it a crucial target for terpenoid production. Secondly, we have shown that the substrate concentration of all MEP pathway enzymes is well below their K_m_, which gives the pathway a high sensitivity of its flux towards substrate changes. Furthermore, we provided a detailed picture of the control of Dxs (Fig. 8) in a production strain, expressing a plasmid-based isoprene synthase and *idi.* Dxs has substantial control over the flux through the metabolite pool of DXP over the entire analyzed expression range (Fig. 6). In contrast, the control over the production of isoprene is high at wild-type *dxs* expression levels and below, but this influence decreases rapidly when the enzyme is overexpressed. The metabolite concentrations of the MEP pathway intermediates gave further insight into this behavior: The concentrations of DXP, MEP and ME-CDP, the first metabolites of the MEP pathway, were linearly dependent on *dxs* expression over the investigated range (Fig. 5) and therefore the concentration control coefficients of Dxs for these metabolites are 1. Likewise, the concentration of MEcPP showed a linear dependence on Dxs levels below the wild type expression level, but a steep increase at higher expression levels with a concentration control coefficient of 2.6 (Fig. 5b). In contrast, the concentration of IPP/DMAPP and the flux towards isoprene showed a saturating behavior, both with a control coefficient of 0.35 (Figs. 4 and 5a) at wild-type *dxs* levels. The steep increase in MEcPP concentration and the decrease in the concentration control coefficient between MEcPP and IPP/DMAPP implies a flux limiting conversion step in the pathway between these metabolites. Neither the catalytic steps between MEP, DXP, ME-CDP and MEcPP, nor between IPP/DMAPP and isoprene seem to limit the flux. The increase in the concentration of DXP and MEcPP correlated with an increase efflux thereof (Fig. 7), which caused the majority of the flux of the MEP pathway to leak into the extracellular space rather than being channeled into isoprene. Therefore, the activity of the enzymes converting MEcPP into IPP/DMAPP (i.e., IspG and IspH) may well have to be increased to reach higher fluxes in MEP pathway towards terpenoids in cell factories. However, simple overexpression of IspG and IspH did not increase the flux towards terpenoids in this work nor did it in other works [17]. IspG needs several cofactors for its activity [57, 58], and this may affect the ability of the enzyme to function as expected when overexpressed.

Therefore, besides identifying Idi and Dxs as major flux controlling steps and vital metabolic engineering targets for microbial terpenoid production, we show that further research is required to determine the precise mechanism of IspG and potentially IspH to allow higher activity thereof to the end of full exploitation of the MEP pathway for the efficient production of terpenoids.

To our knowledge, this is the first use of recombineering combined with targeted proteomics and metabolomics for the precise metabolic control analysis of a specific pathway.

## Supplementary information

**Additional file 1.** Additional tables and figures.

## Data Availability

The datasets used and/or analysed during the current study are available from the corresponding author on reasonable request.

## References

[1] Koksal M, Hu H, Coates RM, Peters RJ, Christianson DW (2011). Structure and mechanism of the diterpene cyclase ent-copalyl diphosphate synthase. Nat Chem Biol.

[2] Kingston DG (2007). The shape of things to come: structural and synthetic studies of taxol and related compounds. Phytochemistry.

[3] White NJ (1997). Assessment of the pharmacodynamic properties of antimalarial drugs in vivo. Antimicrob Agents Chemother.

[4] Wang C, Yoon SH, Jang HJ, Chung YR, Kim JY, Choi ES (2011). Metabolic engineering of *Escherichia coli* for alpha-farnesene production. Metab Eng.

[5] McGarvey DJ, Croteau R (1995). Terpenoid metabolism. Plant Cell.

[6] Morrone D, Lowry L, Determan MK, Hershey DM, Xu M, Peters RJ (2010). Increasing diterpene yield with a modular metabolic engineering system in *E. coli*: comparison of MEV and MEP isoprenoid precursor pathway engineering. Appl Microbiol Biotechnol.

[7] Whited GM, Feher FJ, Benko DA, Cervin MA, Chotani GK, McAuliffe JC (2010). Technological update: development of a gas-phase bioprocess for isoprene-monomer production using metabolic pathway engineering. Ind Biotechnol.

[8] Wang HH, Isaacs FJ, Carr PA, Sun ZZ, Xu G, Forest CR (2009). Programming cells by multiplex genome engineering and accelerated evolution. Nature.

[9] Ye L, Lv X, Yu H (2016). Engineering microbes for isoprene production. Metab Eng.

[10] Jawaid S, Seidle H, Zhou W, Abdirahman H, Abadeer M, Hix JH (2009). Kinetic characterization and phosphoregulation of the *Francisella tularensis* 1-deoxy-d-xylulose 5-phosphate reductoisomerase (MEP synthase). PLoS ONE.

[11] Tsang A, Seidle H, Jawaid S, Zhou W, Smith C, Couch RD (2011). *Francisella tularensis* 2-C-methyl-d-erythritol 4-phosphate cytidylyltransferase: kinetic characterization and phosphoregulation. PLoS ONE.

[12] Bitok JK, Meyers CF (2012). 2C-Methyl-d-erythritol 4-phosphate enhances and sustains cyclodiphosphate synthase IspF activity. ACS Chem Biol.

[13] Banerjee A, Wu Y, Banerjee R, Li Y, Yan H, Sharkey TD (2013). Feedback inhibition of deoxy-d-xylulose-5-phosphate synthase regulates the methylerythritol 4-phosphate pathway. J Biol Chem.

[14] Zhou K, Zou R, Stephanopoulos G, Too HP (2012). Metabolite profiling identified methylerythritol cyclodiphosphate efflux as a limiting step in microbial isoprenoid production. PLoS ONE.

[15] Steinbüchel A (2003). Production of rubber-like polymers by microorganisms. Curr Opin Microbiol.

[16] Ajikumar PK, Xiao WH, Tyo KE, Wang Y, Simeon F, Leonard E (2010). Isoprenoid pathway optimization for Taxol precursor overproduction in *Escherichia coli*. Science.

[17] Zou R, Zhou K, Stephanopoulos G, Too HP (2013). Combinatorial engineering of 1-deoxy-d-xylulose 5-phosphate pathway using cross-lapping in vitro assembly (CLIVA) method. PLoS ONE.

[18] Zhao Y, Yang J, Qin B, Li Y, Sun Y, Su S (2011). Biosynthesis of isoprene in *Escherichia coli* via methylerythritol phosphate (MEP) pathway. Appl Microbiol Biotechnol.

[19] Zurbriggen A, Kirst H, Melis A (2012). Isoprene production via the mevalonic acid pathway in *Escherichia coli* (Bacteria). BioEnergy Res.

[20] Lv X, Xu H, Yu H (2013). Significantly enhanced production of isoprene by ordered coexpression of genes dxs, dxr, and idi in *Escherichia coli*. Appl Microbiol Biotechnol.

[21] Liu H, Sun Y, Ramos KRM, Nisola GM, Valdehuesa KNG, Lee WK (2013). Combination of Entner–Doudoroff pathway with MEP increases isoprene production in engineered *Escherichia coli*. PLoS ONE.

[22] Partow S, Siewers V, Daviet L, Schalk M, Nielsen J (2012). Reconstruction and evaluation of the synthetic bacterial MEP pathway in *Saccharomyces cerevisiae*. PLoS ONE.

[23] Fell DA (1992). Metabolic control analysis: a survey of its theoretical and experimental development. Biochem J..

[24] Sambrook J, Russell DW (2001). Molecular cloning a laboratory manual.

[25] Datta S, Costantino N, Court DL (2006). A set of recombineering plasmids for gram-negative bacteria. Gene.

[26] Bongers M, Chrysanthopoulos PK, Behrendorff JBYH, Hodson MP, Vickers CE, Nielsen LK (2015). Systems analysis of methylerythritol-phosphate pathway flux in *E. coli*: insights into the role of oxidative stress and the validity of lycopene as an isoprenoid reporter metabolite. Microb Cell Fact.

[27] Rabinowitz JD, Kimball E (2007). Acidic acetonitrile for cellular metabolome extraction from *Escherichia coli*. Anal Chem.

[28] Volkmer B, Heinemann M (2011). Condition-dependent cell volume and concentration of *Escherichia coli* to facilitate data conversion for systems biology modeling. PLoS ONE.

[29] Gaida SM, Liedtke A, Jentges AHW, Engels B, Jennewein S (2016). Metabolic engineering of *Clostridium cellulolyticum* for the production of n-butanol from crystalline cellulose. Microb Cell Fact.

[30] MacLean B, Tomazela DM, Shulman N, Chambers M, Finney GL, Frewen B (2010). Skyline: an open source document editor for creating and analyzing targeted proteomics experiments. Bioinformatics.

[31] Altschul SF, Madden TL, Schäffer AA, Zhang J, Zhang Z, Miller W (1997). Gapped BLAST and PSI-BLAST: a new generation of protein database search programs. Nucleic Acids Res.

[32] Perkins DN, Pappin DJC, Creasy DM, Cottrell JS (1999). Probability-based protein identification by searching sequence databases using mass spectrometry data. Electrophoresis.

[33] Batth TS, Singh P, Ramakrishnan VR, Sousa MM, Chan LJ, Tran HM (2014). A targeted proteomics toolkit for high-throughput absolute quantification of *Escherichia coli* proteins. Metab Eng.

[34] Wright LP, Rohwer JM, Ghirardo A, Hammerbacher A, Ortiz-Alcaide M, Raguschke B (2014). Deoxyxylulose 5-phosphate synthase controls flux through the methylerythritol 4-phosphate pathway in Arabidopsis. Plant Physiol.

[35] Young JD, Shastri AA, Stephanopoulos G, Morgan JA (2011). Mapping photoautotrophic metabolism with isotopically nonstationary (13)C flux analysis. Metab Eng.

[36] Hahn FM, Hurlburt AP, Poulter CD (1999). *Escherichia coli* open reading frame 696 is idi, a nonessential gene encoding isopentenyl diphosphate isomerase. J Bacteriol.

[37] Rohdich F, Hecht S, Gärtner K, Adam P, Krieger C, Amslinger S (2002). Studies on the nonmevalonate terpene biosynthetic pathway: metabolic role of IspH (LytB) protein. Proc Natl Acad Sci.

[38] Garrido-Franco M (2003). Pyridoxine 5′-phosphate synthase: de novo synthesis of vitamin B6 and beyond. Biochim Biophys Acta.

[39] Taylor SV, Kelleher NL, Kinsland C, Chiu HJ, Costello CA, Backstrom AD (1998). Thiamin biosynthesis in *Escherichia coli* Identification of ThiS thiocarboxylate as the immediate sulfur donor in the thiazole formation. J Biol Chem.

[40] Gao X, Gao F, Liu D, Zhang H, Nie X, Yang C (2016). Engineering the methylerythritol phosphate pathway in cyanobacteria for photosynthetic isoprene production from CO_2_. Energy Environ Sci.

[41] Brammer LA, Meyers CF (2009). Revealing substrate promiscuity of 1-deoxy-d-xylulose 5-phosphate synthase. Org Lett.

[42] Kuzuyama T, Takahashi S, Takagi M, Seto H (2000). Characterization of 1-deoxy-d-xylulose 5-phosphate reductoisomerase, an enzyme involved in isopentenyl diphosphate biosynthesis, and identification of its catalytic amino acid residues. J Biol Chem.

[43] Koppisch AT, Fox DT, Blagg BS, Poulter CDE (2002). *E. coli* MEP synthase: steady-state kinetic analysis and substrate binding. Biochemistry.

[44] Cane DE, Chow C, Lillo A, Kang I (2001). Molecular cloning, expression and characterization of the first three genes in the mevalonate-independent isoprenoid pathway in *Streptomyces coelicolor*. Bioorg Med Chem.

[45] Moreno-Sanchez R, Saavedra E, Rodriguez-Enriquez S, Olin-Sandoval V (2008). Metabolic control analysis: a tool for designing strategies to manipulate metabolic pathways. J Biomed Biotechnol.

[46] Farmer WR, Liao JC (2001). Precursor balancing for metabolic engineering of lycopene production in *Escherichia coli*. Biotechnol Prog.

[47] Richard SB, Lillo AM, Tetzlaff CN, Bowman ME, Noel JP, Cane DE (2004). Kinetic analysis of *Escherichia coli* 2-C-methyl-d-erythritol-4-phosphate cytidyltransferase, wild type and mutants, reveals roles of active site amino acids. Biochemistry.

[48] Bernal C, Palacin C, Boronat A, Imperial S (2005). A colorimetric assay for the determination of 4-diphosphocytidyl-2-C-methyl-d-erythritol 4-phosphate synthase activity. Anal Biochem.

[49] Xiao Y, Zahariou G, Sanakis Y, Liu P (2009). IspG enzyme activity in the deoxyxylulose phosphate pathway: roles of the iron-sulfur cluster. Biochemistry.

[50] Xiao Y, Chu L, Sanakis Y, Liu P (2009). Revisiting the IspH catalytic system in the deoxyxylulose phosphate pathway: achieving high activity. J Am Chem Soc.

[51] Jonnalagadda V, Toth K, Richard JP (2012). Isopentenyl diphosphate isomerase catalyzed reactions in D2O: product release limits the rate of this sluggish enzyme-catalyzed reaction. J Am Chem Soc.

[52] Bennett BD, Kimball EH, Gao M, Osterhout R, Van Dien SJ, Rabinowitz JD (2009). Absolute metabolite concentrations and implied enzyme active site occupancy in *Escherichia coli*. Nat Chem Biol.

[53] Christodoulou D, Link H, Fuhrer T, Kochanowski K, Gerosa L, Sauer U (2018). Reserve flux capacity in the pentose phosphate pathway enables *Escherichia coli*’s rapid response to oxidative stress. Cell Syst.

[54] Bernal C, Mendez E, Terencio J, Boronat A, Imperial S (2005). A spectrophotometric assay for the determination of 4-diphosphocytidyl-2-C-methyl-d-erythritol kinase activity. Anal Biochem.

[55] Zepeck F, Grawert T, Kaiser J, Schramek N, Eisenreich W, Bacher A (2005). Biosynthesis of isoprenoids. Purification and properties of IspG protein from *Escherichia coli*. J Org Chem.

[56] Fell DA (1998). Increasing the flux in metabolic pathways: a metabolic control analysis perspective. Biotechnol Bioeng.

[57] Grawert T, Kaiser J, Zepeck F, Laupitz R, Hecht S, Amslinger S (2004). IspH protein of *Escherichia coli*: studies on iron–sulfur cluster implementation and catalysis. J Am Chem Soc.

[58] Puan KJ, Wang H, Dairi T, Kuzuyama T, Morita CT (2005). *fldA* is an essential gene required in the 2-C-methyl-d-erythritol 4-phosphate pathway for isoprenoid biosynthesis. FEBS Lett.

