# Supplementary material for: Investigation of the methylerythritol 4-phosphate pathway for microbial terpenoid production through metabolic control analysis
Source: Microb Cell Fact. 2019 Nov 5;18:192. doi: 10.1186/s12934-019-1235-5 (PMC6833178; doi:10.1186/s12934-019-1235-5)
Supplement: Supplementary file 1 — Additional file 1. Additional tables and figures. [file 12934_2019_1235_MOESM1_ESM.docx]

## Additional data

### Sequence of the isoprene synthase gene *isp*S

Table S1 Sequence of the isoprene synthase from *P. alba*, codon optimized for *E. coli*

| ATGGAAGCTCGTCGTTCTGCTAACTACGAACCGAACTCTTGGGACTACGACTACCTGCTGTCTTCTGACACCGACGAATCTATCGAAGTTTACAAAGACAAAGCTAAAAAACTGGAAGCTGAAGTTCGTCGTGAAATCAACAACGAAAAAGCTGAATTCCTGACCCTGCTGGAACTGATCGACAACGTTCAGCGTCTGGGTCTGGGTTACCGTTTCGAATCTGACATCCGTGGTGCTCTGGACCGTTTCGTTTCTTCTGGTGGTTTCGACGCTGTTACCAAAACCTCTCTGCACGGTACCGCTCTGTCTTTCCGTCTGCTGCGTCAGCACGGTTTCGAAGTTTCTCAGGAAGCTTTCTCTGGTTTCAAAGACCAGAACGGTAACTTCCTGGAAAACCTGAAAGAAGACATCAAAGCTATCCTGTCTCTGTACGAAGCTTCTTTCCTGGCTCTGGAAGGTGAAAACATCCTGGACGAAGCTAAAGTTTTCGCTATCTCTCACCTGAAAGAACTGTCTGAAGAAAAAATCGGTAAAGAACTGGCTGAACAGGTTAACCACGCTCTGGAACTGCCGCTGCACCGTCGTACCCAGCGTCTGGAAGCTGTTTGGTCTATCGAAGCTTACCGTAAAAAAGAAGACGCTAACCAGGTTCTGCTGGAACTGGCTATCCTGGACTACAACATGATCCAGTCTGTTTACCAGCGTGACCTGCGTGAAACCTCTCGTTGGTGGCGTCGTGTTGGTCTGGCTACCAAACTGCACTTCGCTCGTGACCGTCTGATCGAATCTTTCTACTGGGCTGTTGGTGTTGCTTTCGAACCGCAGTACTCTGACTGCCGTAACTCTGTTGCTAAAATGTTCTCTTTCGTTACCATCATCGACGACATCTACGACGTTTACGGTACCCTGGACGAACTGGAACTGTTCACCGACGCTGTTGAACGTTGGGACGTTAACGCTATCAACGACCTGCCGGACTACATGAAACTGTGCTTCCTGGCTCTGTACAACACCATCAACGAAATCGCTTACGACAACCTGAAAGACAAAGGTGAAAACATCCTGCCGTACCTGACCAAAGCTTGGGCTGACCTGTGCAACGCTTTCCTGCAGGAAGCTAAATGGCTGTACAACAAATCTACCCCGACCTTCGACGACTACTTCGGTAACGCTTGGAAATCTTCTTCTGGTCCGCTGCAGCTGGTTTTCGCTTACTTCGCTGTTGTTCAGAACATCAAAAAAGAAGAAATCGAAAACCTGCAGAAATACCACGACACCATCTCTCGTCCGTCTCACATCTTCCGTCTGTGCAACGACCTGGCTTCTGCTTCTGCTGAAATCGCTCGTGGTGAAACCGCTAACTCTGTTTCTTGCTACATGCGTACCAAAGGTATCTCTGAAGAACTGGCTACCGAATCTGTTATGAACCTGATCGACGAAACCTGGAAAAAAATGAACAAAGAAAAACTGGGTGGTTCTCTGTTCGCTAAACCGTTCGTTGAAACCGCTATCAACCTGGCTCGTCAGTCTCACTGCACCTACCACAACGGTGACGCTCACACCTCTCCGGACGAACTGACCCGTAAACGTGTTCTGTCTGTTATCACCGAACCGATCCTGCCGTTCGAACGT |
| --- |

### LC-MS/MS settings for targeted metabolomics

MS data were acquired using Analyst v1.6.2 software and a scheduled MRM algorithm. The detection window was individually set for each analyte, but was at least three times the peak width. MultiQuant v3.0.1 was used for the integration of peak area counts for the quantification and evaluation of the spectra.

Table S2 Mass spectrometer settings for targeted metabolomics and targeted proteomics. Parameters were optimized for the flow rate and buffer used for separation.

| Parameter | Value for metabolite analysis | Value for peptide  analysis |
| --- | --- | --- |
| Ion source | Electrospray ionization | Electrospray ionization |
| Curtain gas [psig] | 10 | 32 |
| Collision gas | High | Medium |
| Ion spray voltage [v] | -3000 | 5000 |
| Temperature [°c] | 700 | 550 |
| Ion source gas 1 [psig] | 40 | 44 |
| Ion source gas 2 [psig] | 30 | 32 |
| Entrance potential [v] | Compound dependent | 10 |
| Collision cell exit potential [v] | Compound dependent | 12 |

Fig. S1 Chromatogram of MEP intermediates identified in *E. coli* mdxs7 (pCOLA::IspS-idi) by LC-MS/MS, normalized to their maximum intensity. All peaks are baseline separated. Tailing of IPP/DMAPP is visible. Each color shows the MRM of the depicted metabolite. IPP and DMAPP could not be separated. Raw data are presented without processing (smoothing).

Table S3 Parameters for the detection of MEP pathway intermediates and their isotopes by LC-MS/MS analysis. All metabolites were compared to commercial standards, except MEP-CDP. For MEP-CDP, the fragments were taken from Li and Sharkey (1) and then optimized from an injected *E. coli* extract. Abbreviations: DP = declustering potential; EP = entrance potential; CE = collision energy; CXP = collision exit potential; RT = retention time)

| **Compound** | **Parent ion (Hill notation; linear formula for isotopologs)** | **Parent mass [m/z]** | **Product formula (Hill notation)** | **Product mass [m/z]** | **DP[V]** | **EP[V]** | **CE[V]** | **CXP[V]** | **RT[min]** |
| --- | --- | --- | --- | --- | --- | --- | --- | --- | --- |
| Pyruvate | CH_3_COCO_2_^-^ | 87 | CH_3_CO^-^ | 43 | -40 | -10 | -10 | -7 | 7.48 |
| Pyruvate-U-^13^C_3_ | ^13^CH_3_^13^CO^13^CO_2_^-^ | 90 | ^13^CH_3_^13^CO^-^ | 45 | -40 | -10 | -10 | -7 | 7.48 |
| GAP | C_3_H_6_O_6_P^--^ | 169 | H_2_PO_4_^-^ | 97 | -5 | -10 | -14 | -9 | 6.17 |
| GAP-U-^13^C | C_3_H_6_O_6_P^-^ | 172 | H_2_PO_4_^-^ | 97 | -5 | -10 | -14 | -9 | 6.17 |
| MEP | C_5_H_12_O_7_P^-^ | 215 | PO_3_^-^ | 79 | -5 | -10 | -60 | -17 | 6.25 |
| MEP-U^13^C_5_ | C_5_H_12_O_7_P^-^ | 220 | PO_3_^-^ | 79 | -5 | -10 | -60 | -17 | 6.25 |
| DXP | C_5_H_10_O_7_P^­-^ | 213 | H_2_PO_4_^-^ | 97 | -25 | -10 | -14 | -9 | 6.53 |
| DXP-^13^C_1_ | C_5_H_10_O_7_P^­-^ | 214 | H_2_PO_4_^-^ | 97 | -25 | -10 | -14 | -9 | 6.53 |
| DXP-^13^C_2_ | C_5_H_10_O_7_P^­-^ | 215 | H_2_PO_4_^-^ | 97 | -25 | -10 | -14 | -9 | 6.53 |
| DXP-^13^C_3_ | C_5_H_10_O_7_P^­-^ | 216 | H_2_PO_4_^-^ | 97 | -25 | -10 | -14 | -9 | 6.53 |
| DXP-^13^C_4_ | C_5_H_10_O_7_P^­-^ | 217 | H_2_PO_4_^-^ | 97 | -25 | -10 | -14 | -9 | 6.53 |
| DXP-U^13^C_5_ | C_5_H_10_O_7_P^­-^ | 218 | H_2_PO_4_^-^ | 97 | -25 | -10 | -14 | -9 | 6.53 |
| HMBPP | C_5_H_11_O_8_P_2_^­-^ | 261 | PO_3_^-^ | 79 | -25 | -10 | -32 | -9 | 8.33 |
| HMBPP-U^13^C_5_ | C_5_H_11_O_8_P_2_^­-^ | 266 | PO_3_^-^ | 79 | -25 | -10 | -32 | -9 | 8.33 |
| CDP-ME | \| C_14_H_24_N_3_O_14_P_2_^­-^ \| \| --- \| | 520 | C_9_H_13_N_3_O_8_P^-^ | 322 | -100 | -10 | -30 | -21 | 7.43 |
| CDP-ME-^13^C_5_ | C_14_H_24_N_3_O_14_P_2_^­-^ | 534 | C_9_H_13_N_3_O_8_P^-^ | 322 | -100 | -10 | -30 | -21 | 7.43 |
| CDP-MEP | C_14_H_25_N_3_O_17_P_3_^­-^ | 600 | C_5_H_11_O_9_P_2_^-^ | 277 | -100 | -10 | -30 | -21 | 10.15 |
| CDP-MEP-U^13^C_5_ | C_14_H_25_N_3_O_17_P_3_^­-^ | 614 | C_5_H_11_O_9_P_2_^-^ | 282 | -100 | -10 | -30 | -21 | 10.15 |
| MECPP | C_5_H_11_O_9_P_2_^-^ | 277 | PO_3_^-^ | 79 | -5 | -10 | -66 | -9 | 7.86 |
| MECPP-U^13^C_5_ | C_5_H_11_O_9_P_2_^-^ | 282 | PO_3_^-^ | 79 | -5 | -10 | -66 | -9 | 7.86 |
| IPP/DMAPP | C_5_H_11_O_7_P_2_^-^ | 245 | PO_3_^-^ | 79 | -20 | -10 | -48 | -9 | 11.09 |
| IPP/DMAPP-U^13^C_5_ | C_5_H_11_O_7_P_2_^-^ | 250 | PO_3_^-^ | 79 | -20 | -10 | -48 | -9 | 11.09 |

### Targeted Proteomics

Fig. S2 Chromatogram representing the targeted proteomics analysis of MEP pathway enzymes. The chromatogram shows synthetic standards each of 0.1 pM. Transitions used for quantification are presented in color and the transitions used for qualification are presented in black (Table S4).

Table S4 Transitions and parameters of proteotypic peptides for targeted proteomics analysis. For each peptide, a transition for quantification (quantifier) and a transition for qualification (qualifier) was used.

| **Enzyme Name**  **Quantifier/**  **Qualifier** | **Peptide sequence** | **Fragment Ion** | **Peptide m/z [Da]** | **Transition m/z [Da]** | **Collision energy [V]** | **Declustering potential [V]** | **Collision exit potential [V]** |
| --- | --- | --- | --- | --- | --- | --- | --- |
| Dxs Qual. | LPVLFAIDR | y6 | 522.3 | 734.4 | 39 | 106 | 8 |
| Dxs Quant. | LPVLFAIDR | y5 | 522.3 | 621.3 | 27 | 106 | 32 |
| Dxr Qual. | VVALVAGK | y6 | 378.8 | 487.3 | 17 | 96 | 10 |
| Dxr Quant. | VVALVAGK | y5 | 378.8 | 558.4 | 19 | 101 | 16 |
| IspD Qual. | TGGILAAPVR | y6 | 477.8 | 513.3 | 23 | 101 | 14 |
| IspD Quant. | TGGILAAPVR | y5 | 477.8 | 626.4 | 23 | 101 | 12 |
| IspE Qual. | SIETLLK | y5 | 402.2 | 474.3 | 17 | 96 | 40 |
| IspE Quant. | SIETLLK | y4 | 402.2 | 603.4 | 19 | 96 | 18 |
| IspF Qual. | MLPHIPQMR | y5 | 561.2 | 531.3 | 33 | 101 | 24 |
| IspF Quant. | MLPHIPQMR | y4 | 561.2 | 644.4 | 35 | 101 | 32 |
| IspG Qual. | VGADIVR | y4 | 365.2 | 387.3 | 19 | 96 | 34 |
| IspG Quant. | VGADIVR | y3 | 365.2 | 502.3 | 27 | 91 | 26 |
| IspH Qual. | YVVDSLR | y5 | 426.2 | 490.3 | 19 | 96 | 12 |
| IspH Quant. | YVVDSLR | y4 | 426.2 | 589.3 | 21 | 96 | 24 |
| Idi Qual. | GQLLVTR | y5 | 393.7 | 488.3 | 17 | 91 | 14 |
| Idi Quant. | GQLLVTR | y4 | 393.7 | 601.4 | 19 | 91 | 30 |

### Isoprene production by batch fermentation with *dxs* overexpression

#### Construction of plasmids for overexpression of ispS, idi and dxs

The plasmid pCOLA::IspS-idi was amplified with using forward primer 5′-TCT AAC TAA CCT CCT ATA CTA AGA TGG GG AAT TGT TAT CCG-3′ and reverse primer 5′-TAA CCT AGG CTG CTG CCA CC-3′ to integrate *dxs* at the second multiple cloning site. The *dxs* gene was amplified from the genome of *E. coli* BL21 (DE3) using forward primer 5′-ACA ATT CCC CAT CTT AGT ATA GGA GGT TAG TTA GAA TGA GTT TTG ATA TTG CCA AAT ACC CG-3′ and reverse primer 5′-AGT TAT TGC TCA GCG GTG GCA GCA GCC TAG GTT ATT ATG CCA GCC AGG CCT TGA TTT TGG CTT CC-3′ and the fragments were joined by Gibson assembly.

Table S5 Calculation of the flux through enzymatic steps after branches in the MEP pathway.

| Enzyme | *k*_cat_ [min^-1^] | *K*_m_ [µM] | Reference | Substrate concentration in cell [µM] | Protein copies per cell | Flux per cell [min^-1^] |
| --- | --- | --- | --- | --- | --- | --- |
| Dxs | 250 | 226 | (2) | 10.3 | 64 | 365 |
| Dxr | 5.9 x 10^5^ | 250 | (3) | 16.8 | 32 | 6.34 x 10^5^ |
|  | 1.3 x 10^5^ | 99 |  |  |  | 3.52 x 10^5^ |
|  | 3.1 x 10^5^ | 60 |  |  |  | 8.42 x 10^5^ |
|  | 6.96 x 10^3^ | 115 | (4) |  |  | 16.27 x 10^3^ |
|  | 1.3 x 10^3^ | 720 | (5) |  |  | 4.86 x 10^2^ |

### Data for the *dxs* expression library

Table S6 RBS sequence of the *dxs* expression library.

| ***dxs* mutant #** | **RBS sequence** |
| --- | --- |
| ssDNA | CTADDRRRRRDDDDCTGATG |
| 1- wild type | GTATTAATAGGCCCCTGATG |
| 2 | CTAGGAAGGGATTTCTGATG |
| 3 | CTATGAGAGAGGGGCTGATG |
| 4 | CTATTGAAGGGTTTCTGATG |
| 5 | CTATTGAGATGTGCTGATG |
| 6 | CTAGTAGGGAGAGACTGATG |
| 7 | CTATGAAGAGGTTGCTGATG |
| 8 | CTAGTAGGGAGGTGCTGATG |
| 9 | CTATTGAGAGTTGTCTGATG |
| 10 | CTAAGGGAAGTTTGCTGATG |
| 11 | CTATTGAAAAGTATCTGATG |
| 12 | CTAAGGAAAATGGTCTGATG |
| 13 | CTATTAAGAATATTCTGATG |
| 14 | CTAGAAAGAGTATTCTGATG |
| 15 | CTATAGAAGGATGGCTGATG |

### Data for the *dxr* expression library

Table S7 RBS sequences in the *dxr* expression library.

| ***dxr* mutant #** | **RBS sequence** |
| --- | --- |
| Inv_ssDNA | CAATTDDRRRRRDDDDATTATG |
| 1- wild type | GAATTACATGTGAGAAATTATG |
| 2 | CAATTATGGAAGTGGTCTTATG |
| 3 | CAATTATAAAGATTATATTATG |
| 4 | CAATTTGAAAGGAATAATTATG |
| 5 | CAATTTGAAGGAATAAATTATG |
| 6 | CAATTGTGAAAATATTATTATG |
| 7 | CAATTTGGGGAATGTAATTATG |
| 8 | CAATTATGAGAATGGGATTATG |
| 9 | CAATTTGAGAGAATAAATTATG |
| 10 | CAATTAAGGAGAAAAAATTATG |
| 11 | CAATTTGAGAGGATAGATTATG |
| 12 | CAATTATGGAGAGAATATTATG |
| 13 | CAATTATAAAAGTTAAATTATG |
| 14 | CAATTGTAAGAAATTTATTATG |
| 15 | CAATTTTAGAGATGTTATTATG |

Fig. S3 Dxr content in the mutants of the *dxr* expression library.

### Data for isoprene production assay


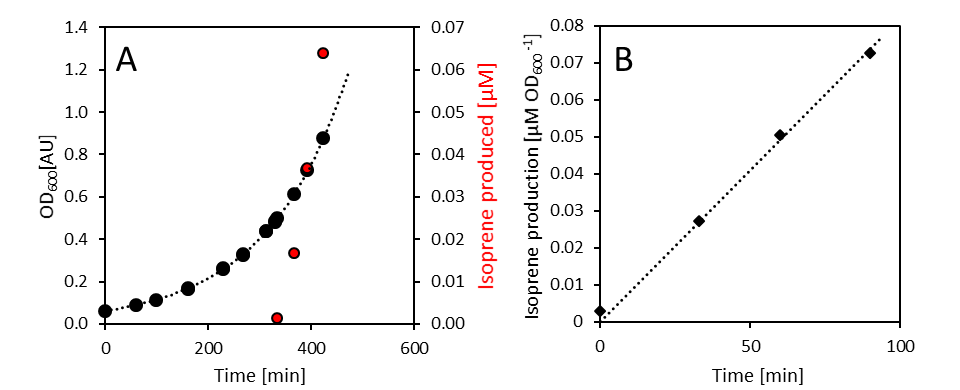


Fig. S4 Data from the isoprene production assay in *E. coli* mdxs6 pCOLA::IspS-idi. (A) The growth of the strain was monitored after induction (•). The growth time-course data were was fitted with an exponential curve. Isoprene production was measured after sealing the samples at OD_600_ = 0.5 (•). (B) After normalization of the isoprene concentration to biomass, isoprene production was plotted against incubation time. The curve was fitted with a linear equation through zero.


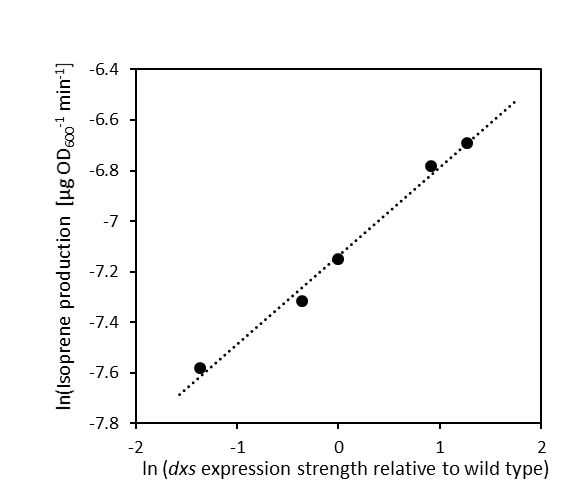


Fig. S5 Dxs control over the production of isoprene. The logarithm of isoprene production is plotted against the logarithm of the relative expression of *dxs* in the expression library. The linear fit has a slope of 0.3505 ($\bar{\boldsymbol{x}}$, n = 3).

#### Isoprene production by batch fermentation

The production of isoprene was quantified in the strains carrying the empty pCOLA-Duet1 plasmid or containing the genes *isp*S, *isp*S and *idi*, or *isp*S, *idi* and *dxs*. The strains were grown in baffled Erlenmeyer flasks in LB medium until the OD_600_ reached 0.5. After adding IPTG to a final concentration of 1 mM, 200 mL of the cultures were transferred to 250-mL serum bottles and sealed immediately. The strains were grown overnight and isoprene was measured in the headspace. No isoprene was detected in the strain with the empty pCOLADuet-1 plasmid. Isoprene production increased from 0.212 ± 0.020 mg OD_600_^-1^L^-1^ in the strain solely expressing *isp*S to 0.465± 0.005 mg OD_600_^-1^ L^-1^ if *idi* was co-expressed. The additional expression of *dxs* increased the yield to 1.443± 0.053 mg OD_600_^-1^ L^-1^.

Fig. S6 Isoprene production by batch fermentation. *E. coli* carrying the plasmids pCOLA, pCOLA::IspS , pCOLA::IspS-idi and pCOLA::IspS-idi,dxs were compared. The cultures were induced at OD_600_ = 0.5 and sealed. The headspace was insufficient to support aerobic growth. The cultures were grown overnight. The data represent biological triplicates ($\bar{\mathbf{x}}$± SD, n = 3).


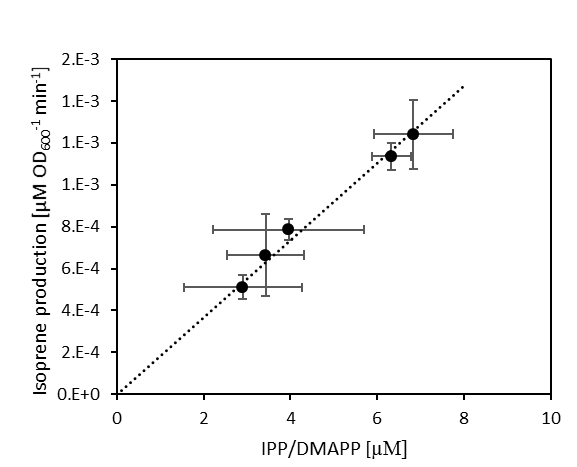


Fig. S7 Isoprene production as a function of intracellular IPP/DMAPP concentration. The graph was fitted with the linear equation through zero with the slope 1.84 x 10^-4^ (R^2^=0.985) ($\bar{\text{x}}$± SD, n = 3).

Table S8 MEP pathway intermediate concentrations in engineered strains with altered *dxs* expression. Values are means of three independent biological replicates and three different time points ± SD at OD_600_ ≈ 0.5 and after an additional 30 min and 60 min.

| **Strains** | **Intermediate concentration [µM]** | | | | |
| --- | --- | --- | --- | --- | --- |
| *E. coli* BL21 (DE3) | **DXP** | **MEP** | **ME-CDP** | **MEcPP** | **IPP/DMAPP** |
| wt pCOLA | 16.8 ± 0.7 | 0.75 ± 0.04 | 0.92 ± 0.02 | 12.2 ± 0.01 | 1.3 ± 0.2 |
| wt pCOLA::IspS-idi | 59.5 ± 0.2 | 3.7 ± 2.1 | 1.7 ± 0.4 | 14 ± 3 | 4 ± 1.7 |
| mdxs2 pCOLA::IspS-idi | 20.8 ± 6.9 | 1.7 ± 0.5 | 1 ± 0.3 | 7.5 ± 2.1 | 2.9 ± 1.4 |
| mdxs5 pCOLA::IspS-idi | 51.5 ± 13.8 | 2.7 ± 0.3 | 1.8 ± 1 | 12.3 ± 6.3 | 3.4 ± 0.9 |
| mdxs6 pCOLA::IspS-idi | 182.1 ± 42.9 | 7.8 ± 3.1 | 4.8 ± 2.8 | 199 ± 63.4 | 6.8 ± 0.4 |
| mdxs7 pCOLA::IspS-idi | 252 ± 6.5 | 13.8 ± 4.1 | 6.2 ± 1.6 | 353.6 ± 56 | 6.3 ± 0.9 |

Fig. S8 Logarithmic plot of the concentration of MEP intermediates as a function of *dxs* expression. The concentration control coefficient is the slope of the curve. All curves were fitted linearly. Red bordered data points are outliers and were not included in the fitting. ●DXP, ●MEP, ●ME-CDP, ●MEcPP and ●IPP/DMAPP. ($\bar{\text{x}}$, n = 3).

Table S9 Control coefficients of Dxs in *E. coli* pCOLA::IspS-idi for MEP pathway intermediates and flux. The concentration control coefficients are calculated from Fig. S8 and the flux control coefficient from Fig. S5. The control coefficient MEcPP‑high represents the concentration control coefficient of Dxs on MEcPP at *dxs* expression levels higher than wild-type, while MEcPP-low refers to *dxs* expression levels below wild-type.

| **Metabolite/Flux** | **Control coefficient** | **Certainty (R² )** |
| --- | --- | --- |
| DXP | 0.95 | 0.999 |
| MEP | 0.82 | 0.999 |
| ME-CDP | 0.71 | 0.995 |
| MEcPP-low | 0.46 | 0.997 |
| MEcPP-high | 2.6 | 0.989 |
| IPP/DMAPP | 0.45 | 0.990 |
| Isoprene production flux | 0.35 | 0.989 |
| MEcPP export | 2.0 | 0.998 |
| DXP export | 1.3 | 0.970 |
| Flux towards extracellular DXP, MEcPP and isoprene | 1.63 | 0.996 |
| Flux through DXP | 0.65 | 0.982 |


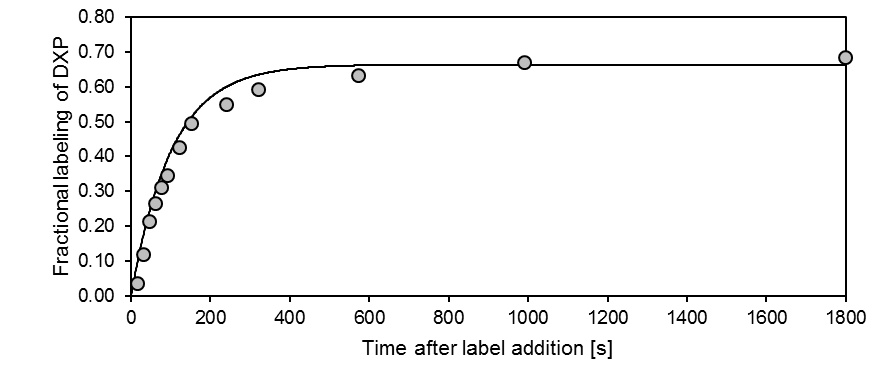


Fig. S9 Example of a time course of ^13^C label incorporation into the DXP metabolite pool. Here shown is the data of *E. coli* expressing isoprene synthase and isopentenyl isomerase from the plasmid pCOLA::IspS-idi. The kinetic rate constant was calculated based on fitting the data to an exponential rise to maximum (black line) (see Materials and Methods).

References

1. Li Z, Sharkey TD. Metabolic profiling of the methylerythritol phosphate pathway reveals the source of post-illumination isoprene burst from leaves. Plant Cell Environ. 2013;36(2):429-37.

2. Brammer LA, Smith JM, Wade H, Meyers CF. 1-Deoxy-D-xylulose 5-phosphate synthase catalyzes a novel random sequential mechanism. J Biol Chem. 2011;286(42):36522-31.

3. Kuzuyama T, Takahashi S, Takagi M, Seto H. Characterization of 1-deoxy-D-xylulose 5-phosphate reductoisomerase, an enzyme involved in isopentenyl diphosphate biosynthesis, and identification of its catalytic amino acid residues. J Biol Chem. 2000;275(26):19928-32.

4. Koppisch AT, Fox DT, Blagg BS, Poulter CD. *E. coli* MEP synthase: steady-state kinetic analysis and substrate binding. Biochemistry. 2002;41(1):236-43.

5. Cane DE, Chow C, Lillo A, Kang I. Molecular cloning, expression and characterization of the first three genes in the mevalonate-independent isoprenoid pathway in *Streptomyces coelicolor*. Bioorg Med Chem. 2001;9(6):1467-77.
